# Z-folding aircraft electromagnetic scattering analysis based on hybrid grid matrix transformation

**DOI:** 10.1038/s41598-022-08385-9

**Published:** 2022-03-15

**Authors:** Zeyang Zhou, Jun Huang

**Affiliations:** School of Aeronautic Science and Engineering, Beihang University, Beijing, 100191 China

**Keywords:** Aerospace engineering, Optical physics

## Abstract

To study the electromagnetic scattering characteristics of a morphing aircraft with Z-folding wings, a method of hybrid grid matrix transformation (HGMT) is presented. The radar cross-section (RCS) of the aircraft in the four Z-folding modes is calculated and analyzed. When considering the deflection of the outer wing separately, the RCS of the wing under the head and side azimuth shows obvious dynamic characteristics, while the peak and fluctuation range are quite different. When the mid wing and the outer wing are deflected upwards together, the RCS of the aircraft under the positive side direction could be significantly reduced. When the mid wing deflects upward and the outer wing remains level, the peak of the side RCS of the aircraft is slightly reduced. When the mid wing deflects upwards and the outer wing deflects downwards, this peak indicator is further reduced, while the local fluctuation of the side RCS of the aircraft is increased. The HGMT method is effective to study the electromagnetic scattering characteristics of the Z-folding aircraft.

## Introduction

With the increasing demand for multi-tasking, covert investigation and efficient flight, research on the aerodynamics, aero elasticity, stealth requirements and material structure of morphing aircraft has gained a lot of attention^1,2^. Common variants include changes in wing length, increased sweep angle, variable area, and wing folding^3,4^. The deformation causes a direct change in the shape of the aircraft, which in turn affects the aerodynamic characteristics and stealth performance^5^. Facing the complex radar detection threats in the future battlefield, the research on the electromagnetic scattering characteristics of morphing aircraft has important practical significance and engineering value.

In the early design and application, the droppable nose of the aircraft provided the pilot with a good field of vision during the take-off and landing phases. Other discrete deformations included active elastic wings, variable wingspan and retractable landing gear. An aircraft with foldable wings was presented, which could switch between long-term flight and high-speed movement modes^6,7^, at the same time, the stealth design of aircraft was also considered. The emergence of variant technology was expected to improve flight performance, expand the flight envelope, replace the traditional control surface and improve stealth performance^8,9^. A neutral point was repositioned on the opposite wing, which was close to the hinge point of the wing above the actual center of gravity^10^. According to the needs of the flying mission at the time, the Z-shaped wings of the morphing aircraft could be folded into different configurations. Unstructured grids were used to discretize object shapes, and the flow field of the aircraft after the wings were folded is solved^11,12^, while different folding angles made the ability of the wing to deflect the incident wave different. A bionic bird-wing folding aircraft was presented, where the outer end of the wing could be contracted and expanded, resulting in a change in the plane shape of the wing^13^. Using high-speed video and flow field to measure the movement of the wing, when the stroke amplitude was increased, there was a "flapping and throwing" action on the outside of the wing^14^. A combined deformation design was investigated in detail, including the extension of the outer end of the wing and the increase in the sweep angle^15^. It was worth noting that the change of the wingspan would affect the illumination area under the incident wave from the head, and the change of the sweep angle would affect the deflection effect of the given incident wave.

The aerodynamic and stealth characteristics of the deformed aircraft would be changed, and how the continuous deformation process dynamically affected electromagnetic scattering was worth exploring. When the sweep angle of the wings of the aircraft increased, the projection of the entire machine in the direction of the body axis was constantly decreasing^16^. Research on wing deformation, active elasticity and smart wing further promoted the development of variant aircraft technology^17^. High-precision unstructured grids were used to process the target surface, and physical optics (PO) and physical theory of diffraction (PTD) were used to calculate the radar cross section^18^. The Z-shaped wing was designed and studied, and then used on high-altitude solar-powered morphing aircraft^19,20^, while variation of electromagnetic scattering intensity on the surface of the panel at the outer end of the large deformation wing would be very obvious. The ranking factor was used to filter an optimal solution for the stealth design of a flying wing exhaust system^21^. The aerodynamic lift, drag and velocity distributions of the variant aircraft in the two sweep modes were analyzed and discussed^22^. When there were geometrically complex scattering obstacles, ray tracing became expensive or difficult to handle^23,24^. The measurement algorithm of the wing folding angle based on the three-axis accelerometer was designed, and the attitude of the fuselage and the wing relative to the reference coordinate system was accurately calculated^25^. The multi-rotor dynamic scattering calculation method was presented to solve the radar stealth characteristic change of the compound helicopter^26^. The control problem of a variable-swept wing deformed aircraft was studied, where the nonlinear switching system and adaptive dynamic programming were presented^27^. Damage sensing and self-healing magnetic polymer composites had been studied. This new collaborative method could be applied to the deformation applications of adaptive wing structures^28,29^. Compared with the high-speed rotating rotor, the folding movement of the Z-shaped wing could be regarded as a slow small-angle rotation movement.

In general, the research on morphing aircraft technology has made substantial progress in many aspects, including aeroelastic analysis, active torsion, and sweep angle changes, folding wings, dynamic models, sliding skin and control technology. For Z-shaped wings, the folding of the middle and outer wings will increase the side projection of the aircraft, which will adversely affect its stealth characteristics, while these effects and the dynamic changes of the radar cross section still need to be explored and studied. Therefore, this article attempts to present a hybrid grid matrix transformation method to evaluate the RCS changes brought to the aircraft when the Z-wing is folded. The dynamic effects of multiple folding modes on the electromagnetic scattering characteristics of aircraft will be investigated and discussed. Studying the dynamic scattering of the Z-folding wing is of great importance and engineering value for the stealth design and development of morphing aircraft.

In this manuscript, the HGMT method is presented in “Hybrid grid matrix transformation”. Model of the morphing aircraft is given in “Model”. Related results of aircraft RCS are discussed in “Results and discussion”. Finally, the full text is summarized.

## Hybrid grid matrix transformation

**Figure 1:**
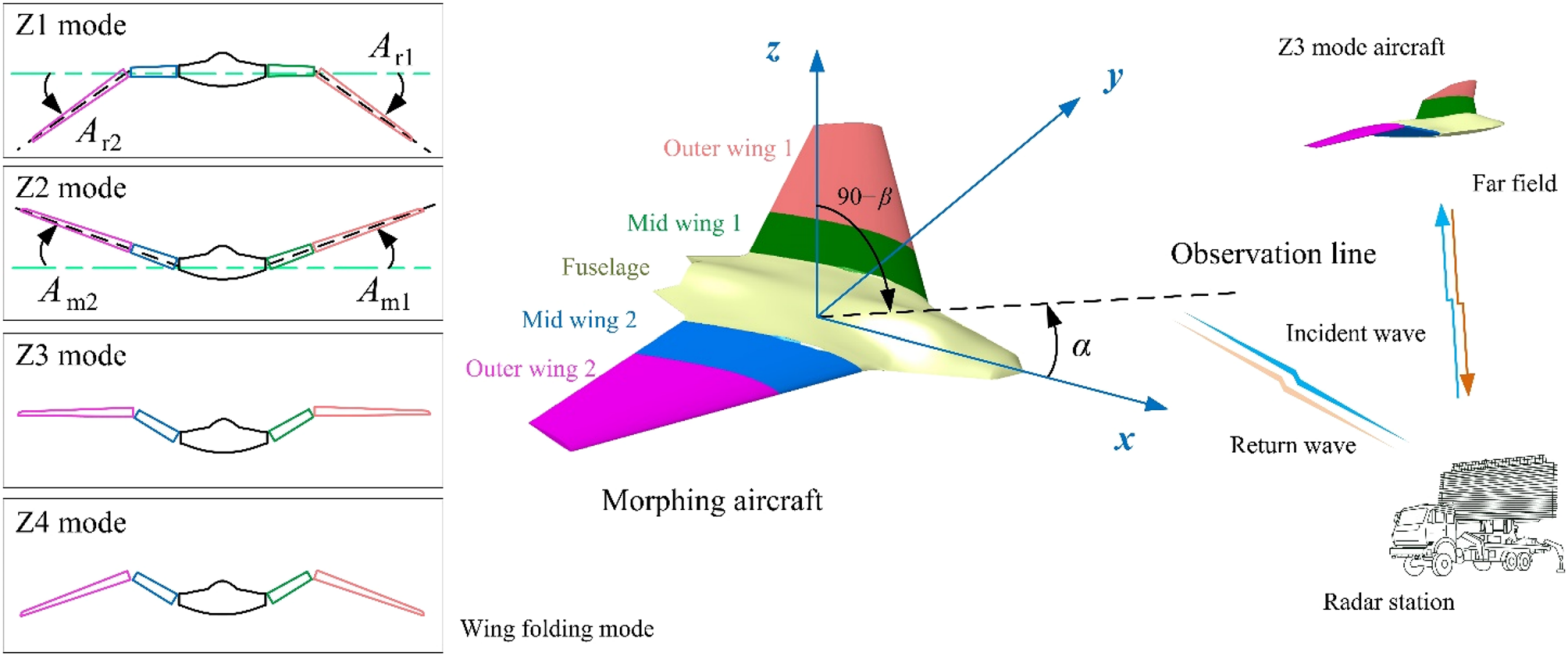
Schematic of dynamic scattering of the morphing aircraft with Z-folding wings.

The schematic diagram of the dynamic electromagnetic scattering of the aircraft in different folding modes is shown in Fig. 1, where *A*_r1_ is the deflection angle of outer wing 1, *A*_m1_ is the deflection angle of mid wing 1, *α* represents the azimuth angle between the radar station and the aircraft, *β* is elevation angle between the radar station and the aircraft, *A*_r2_ is the deflection angle of outer wing 2, *A*_m2_ is the deflection angle of mid wing 2. In the current coordinate system, the middle wing remains level and the outer wing deflects downward when the aircraft is flying in Z1 mode. For Z2 mode, the outer wing is fixedly attached to the middle wing and deflects upwards together. In Z3 mode, the middle wing deflects upwards while the outer wing remains level. When the aircraft is flying in Z4 mode, the middle section wing deflects upward and the outer section wing deflects downward.

### Hybrid grid matrix

In the current observation field, the grid matrix of the aircraft could be expressed as a hybrid form of the following matrices:

1 $${\varvec{M}}_{{\text{a}}}^{{}} \left( {m_{{\text{a}}} \left( t \right)} \right) = \left[ {{\varvec{M}}_{{{\text{r1}}}}^{{}} \left( {m_{{{\text{r}}1}} \left( t \right)} \right),{\varvec{M}}_{{{\text{r2}}}}^{{}} \left( {m_{{{\text{r2}}}} \left( t \right)} \right),{\varvec{M}}_{{{\text{w1}}}}^{{}} \left( {m_{{{\text{w}}1}} \left( t \right)} \right),{\varvec{M}}_{{{\text{w1}}}}^{{}} \left( {m_{{{\text{w}}2}} \left( t \right)} \right),{\varvec{M}}_{{\text{f}}}^{{}} \left( {m_{{\text{f}}} \left( t \right)} \right)} \right]$$

where *t* is time, *m*_a_ represents the aircraft model, *m*_w1_ is the model of mid wing 1, *m*_w2_ is the model of mid wing 2, *m*_f_ is the model of fuselage, ***M***_a_ is the grid coordinate matrix of *m*_a_, *m*_r1_ is the model of outer wing 1, ***M***_r1_ is the grid coordinate matrix of outer wing 1, *m*_r2_ is the model of outer wing 2, ***M***_r2_ is the grid coordinate matrix of outer wing 2, ***M***_w1_ is the grid coordinate matrix of *m*_w1_, ***M***_w2_ is the grid coordinate matrix of *m*_w2_, ***M***_f_ is the grid coordinate matrix of *m*_f_.

When each part of the wing begins to deform and the fuselage remains stationary in the coordinate system, the hybrid grid matrix of the aircraft could be transformed into:

2 $${\varvec{M}}_{{\text{a}}}^{{}} \left( {m_{{\text{a}}} \left( t \right)} \right) = \left[ {{\varvec{M}}_{{{\text{r1}}}}^{{}} \left( {m_{{{\text{r}}1}} \left( t \right)} \right),{\varvec{M}}_{{{\text{r2}}}}^{{}} \left( {m_{{{\text{r2}}}} \left( t \right)} \right),{\varvec{M}}_{{{\text{w1}}}}^{{}} \left( {m_{{{\text{w}}1}} \left( t \right)} \right),{\varvec{M}}_{{{\text{w1}}}}^{{}} \left( {m_{{{\text{w}}2}} \left( t \right)} \right),{\varvec{M}}_{{\text{f}}}^{{}} \left( {m_{{\text{f}}} \left( {t{ = }0} \right)} \right)} \right]$$

At this time, the grid matrix of the aircraft is the combination of the static grid matrix of the fuselage and the dynamic grid matrix of each part of the wing.

When the outer wing and the middle wing deflect together, the local hybrid matrix could be expressed as:

3 $${\varvec{M}}_{{{\text{rw1}}}}^{{}} \left( {m_{{{\text{rw1}}}} \left( t \right)} \right) = \left[ {{\varvec{M}}_{{{\text{r1}}}}^{{}} \left( {m_{{{\text{r}}1}} \left( t \right)} \right),{\varvec{M}}_{{{\text{w1}}}}^{{}} \left( {m_{{{\text{w}}1}} \left( t \right)} \right)} \right]$$

where *m*_rw1_ is a combined model of the mid wing 1 and the outer wing 1, ***M***_rw1_ is the grid coordinate matrix of *m*_rw1_.

Then the hybrid matrix of the aircraft could be updated to:

4 $${\varvec{M}}_{{\text{a}}}^{{}} \left( {m_{{\text{a}}} \left( t \right)} \right) = \left[ {{\varvec{M}}_{{{\text{rw1}}}}^{yx} \left( {m_{{{\text{rw}}1}} \left( t \right)} \right),{\varvec{M}}_{{{\text{rw2}}}}^{yx} \left( {m_{{{\text{rw2}}}} \left( t \right)} \right),{\varvec{M}}_{{\text{f}}}^{{}} \left( {m_{{\text{f}}} \left( t \right)} \right)} \right]$$

where *m*_rw2_ is a combined model of the mid wing 2 and the outer wing 2, ***M***_rw2_ is the grid coordinate matrix of *m*_rw2_, different superscripts represent different transformation operations. The expression of this hybrid matrix is not unique, while the purpose is to integrate the outer wing and middle wing on the left and right sides of the aircraft.

### Matrix transformation during folding deformation

For the Z1 folding mode, when the outer wing 1 deflects downward, its dynamic model could be expressed as:

5 $${\varvec{M}}_{{{\text{r1}}}}^{y} \left( {m_{{{\text{r1}}}} \left( {t = 0} \right)} \right) = {\varvec{M}}_{{\text{r1}}} \left( {y\left( {m_{{{\text{r1}}}} \left( {t = 0} \right)} \right) - Y_{{{\text{r1}}}} } \right)\left| {A_{{{\text{m}}1}} = 0} \right.$$

6 $${\varvec{M}}_{{{\text{r1}}}}^{z} \left( {m_{{{\text{r1}}}} \left( {t = 0} \right)} \right) = {\varvec{M}}_{{{\text{r}}1}}^{y} \left( {z\left( {m_{{{\text{r1}}}} \left( {t = 0} \right)} \right) + Z_{{{\text{r1}}}} } \right)\left| {A_{{{\text{m}}1}} = 0} \right.$$

7 $${\varvec{M}}_{{{\text{r1}}}}^{x} \left( {m_{{{\text{r1}}}} \left( t \right)} \right) = \left[ {\begin{array}{*{20}c} 1 & 0 & 0 \\ 0 & {\cos A_{{{\text{r1}}}} \left( t \right)} & { - \sin A_{{{\text{r1}}}} \left( t \right)} \\ 0 & {\sin A_{{{\text{r1}}}} \left( t \right)} & {\cos A_{{{\text{r1}}}} \left( t \right)} \\ \end{array} } \right] \cdot {\varvec{M}}_{{{\text{r1}}}}^{z} \left( {m_{{{\text{r1}}}} \left( {t = 0} \right)} \right)\left| {\begin{array}{*{20}l} {\begin{array}{*{20}c} {} \\ {} \\ \end{array} } \hfill \\ {A_{{{\text{m1}}}} = 0} \hfill \\ \end{array} } \right.$$

where *Y*_r1_ is the distance from the axis of rotation of the outer wing 1 around the middle wing 1 to the *xz* plane, *Z*_r1_ is the distance from the axis of rotation of the outer wing 1 around the middle wing 1 to the *xy* plane, different superscripts of ***M***_r1_ correspond to the current transformation operation. The wing here is regarded as a rigid body model, and the support on which it deflects is regarded as a rigid body and a stable fixed support. The deflection transformation operation of the outer wing 2 is similar to that of the outer wing 1. Return the transformed outer wing model to the outer end of the middle wing, thus the dynamic matrix of the aircraft could be expressed as:

8 $${\varvec{M}}_{{{\text{a1}}}}^{{}} \left( {m_{{{\text{a1}}}} \left( t \right)} \right) = \left[ {{\varvec{M}}_{{{\text{r1}}}}^{yx} \left( {m_{{{\text{r}}1}} \left( t \right)} \right),{\varvec{M}}_{{{\text{r2}}}}^{yx} \left( {m_{{{\text{r2}}}} \left( t \right)} \right),{\varvec{M}}_{{{\text{w1}}}}^{{}} \left( {m_{{{\text{w}}1}} \left( {t = 0} \right)} \right),{\varvec{M}}_{{{\text{w1}}}}^{{}} \left( {m_{{{\text{w}}2}} \left( {t = 0} \right)} \right),{\varvec{M}}_{{\text{f}}}^{{}} \left( {m_{{\text{f}}} \left( {t{ = }0} \right)} \right)} \right]$$

where *m*_a1_ represents the aircraft model in Z1 folding mode, ***M***_a1_ is the grid coordinate matrix of *m*_a1_.

Under the irradiation of radar waves, the irradiation area on the surface of the aircraft could be extracted as:

9 $$S_{{{\text{a1}}}} \left( t \right) \Leftarrow {\varvec{M}}_{{{\text{a}}1}}^{{}} \left( {m_{{{\text{a}}1}} \left( t \right)} \right)$$

where *S*_a1_ represents the irradiation area on the surface of the aircraft.

For the Z2 folding mode, the middle wing and the outer wing deflect upward together. Then the middle wing 1 and the outer wing 1 are combined into a whole:

10 $${\varvec{M}}_{{{\text{rw1}}}}^{{}} \left( {m_{{{\text{rw1}}}} \left( {t = 0} \right)} \right) = \left[ {{\varvec{M}}_{{{\text{r1}}}}^{{}} \left( {m_{{{\text{r}}1}} \left( {t{ = }0} \right)} \right),{\varvec{M}}_{{{\text{w1}}}}^{{}} \left( {m_{{{\text{w}}1}} \left( {t = 0} \right)} \right)} \right]$$

The deflection process can be described as:

11 $${\varvec{M}}_{{{\text{rw1}}}}^{y} \left( {m_{{{\text{rw1}}}} \left( {t = 0} \right)} \right) = {\varvec{M}}_{{\text{rw1}}} \left( {y\left( {m_{{{\text{rw1}}}} \left( {t = 0} \right)} \right) - Y_{{{\text{w1}}}} } \right)$$

12 $${\varvec{M}}_{{{\text{rw1}}}}^{z} \left( {m_{{{\text{rw1}}}} \left( {t = 0} \right)} \right) = {\varvec{M}}_{{{\text{rw}}1}}^{y} \left( {z\left( {m_{{{\text{rw1}}}} \left( {t = 0} \right)} \right) - Z_{{{\text{w1}}}} } \right)$$

13 $${\varvec{M}}_{{{\text{rw1}}}}^{x} \left( {m_{{{\text{rw1}}}} \left( t \right)} \right) = \left[ {\begin{array}{*{20}c} 1 & 0 & 0 \\ 0 & {\cos A_{{{\text{m1}}}} \left( t \right)} & { - \sin A_{{{\text{m1}}}} \left( t \right)} \\ 0 & {\sin A_{{{\text{m1}}}} \left( t \right)} & {\cos A_{{{\text{m1}}}} \left( t \right)} \\ \end{array} } \right] \cdot {\varvec{M}}_{{{\text{rw1}}}}^{z} \left( {m_{{{\text{rw1}}}} \left( {t = 0} \right)} \right)$$

where *Y*_w1_ is the distance from the axis of the mid wing 1 rotating around the fuselage to the *zx* plane, *Z*_w1_ is the distance from the axis of the mid wing 1 rotating around the fuselage to the *xy* plane. For the other half of the wing, the deflection transformation operation is similar to that of the outer wing 1 and the middle wing 1. Return the transformed wing model to the rotation axis on the side of the fuselage, and the dynamic matrix of the entire aircraft can be updated to:

14 $${\varvec{M}}_{{{\text{a2}}}}^{{}} \left( {m_{{{\text{a2}}}} \left( t \right)} \right) = \left[ {{\varvec{M}}_{{{\text{rw1}}}}^{yx} \left( {m_{{{\text{rw}}1}} \left( t \right)} \right),{\varvec{M}}_{{{\text{rw2}}}}^{yx} \left( {m_{{{\text{rw2}}}} \left( t \right)} \right),{\varvec{M}}_{{\text{f}}}^{{}} \left( {m_{{\text{f}}} \left( {t{ = }0} \right)} \right)} \right]$$

where *m*_a2_ is the aircraft model in Z2 folding mode, ***M***_a2_ is the grid coordinate matrix of *m*_a2_. Thus the irradiation area on the surface of the aircraft can be extracted as:

15 $$S_{{{\text{a2}}}} \left( t \right) \Leftarrow {\varvec{M}}_{{{\text{a2}}}}^{{}} \left( {m_{{{\text{a2}}}} \left( t \right)} \right)$$

where *S*_a2_ represents the irradiation area on the surface of *m*_a2_.

For the Z3 folding mode, the mid wing 1 deflects upwards:

16 $${\varvec{M}}_{{{\text{w1}}}}^{y} \left( {m_{{{\text{w1}}}} \left( {t = 0} \right)} \right) = {\varvec{M}}_{{\text{w1}}} \left( {y\left( {m_{{{\text{w1}}}} \left( {t = 0} \right)} \right) - Y_{{{\text{w1}}}} } \right)$$

17 $${\varvec{M}}_{{{\text{w1}}}}^{z} \left( {m_{{{\text{w1}}}} \left( {t = 0} \right)} \right) = {\varvec{M}}_{{{\text{w}}1}}^{y} \left( {z\left( {m_{{{\text{w1}}}} \left( {t = 0} \right)} \right) - Z_{{{\text{w1}}}} } \right)$$

18 $${\varvec{M}}_{{{\text{w1}}}}^{x} \left( {m_{{{\text{w1}}}} \left( t \right)} \right) = \left[ {\begin{array}{*{20}c} 1 & 0 & 0 \\ 0 & {\cos A_{{{\text{m1}}}} \left( t \right)} & { - \sin A_{{{\text{m1}}}} \left( t \right)} \\ 0 & {\sin A_{{{\text{m1}}}} \left( t \right)} & {\cos A_{{{\text{m1}}}} \left( t \right)} \\ \end{array} } \right] \cdot {\varvec{M}}_{{{\text{w1}}}}^{z} \left( {m_{{{\text{w1}}}} \left( {t = 0} \right)} \right)$$

At the same time, the outer wing 1 follows the outer end of the mid wing 1 to translate:

19 $${\varvec{M}}_{{{\text{r1}}}}^{y} \left( {m_{{{\text{r1}}}} \left( t \right)} \right) = {\varvec{M}}_{{\text{r1}}} \left( {y\left( {m_{{{\text{r1}}}} \left( {t = 0} \right)} \right) + \Delta Y_{{{\text{r1}}}} \left( t \right)} \right)$$

20 $${\varvec{M}}_{{{\text{r1}}}}^{z} \left( {m_{{{\text{r1}}}} \left( t \right)} \right) = {\varvec{M}}_{{{\text{r}}1}}^{y} \left( {z\left( {m_{{{\text{r1}}}} \left( t \right)} \right) + \Delta Z_{{{\text{r1}}}} \left( t \right)} \right)$$

where Δ*Y*_r1_ and Δ*Z*_r1_ represent the translation distance of the outer wing 1 shaft in the *y*-axis and *z*-axis directions, respectively. Noting that:

21 $$\Delta Y_{{{\text{r1}}}} \left( t \right){ = }Y_{{{\text{m1}}}} + R_{0} \cos \left( {A_{{{\text{m1}}}} \left( t \right) - A_{0} } \right) - Y_{{{\text{r1}}}}$$

22 $$\Delta Z_{{{\text{r1}}}} \left( t \right){ = }Z_{{{\text{m1}}}} + R_{0} \sin \left( {A_{{{\text{m1}}}} \left( t \right) - A_{0} } \right) - Z_{{{\text{r1}}}}$$

where *R*_0_ is the distance between *A*_X1_ and *A*_X2_, *A*_X1_ represents the axis of rotation of the outer wing 1 around the mid wing 1, *A*_X2_ is the axis of rotation of the mid wing 1 around the fuselage. *A*_0_ is the angle between the line connecting the projection points of *A*_X1_ and *A*_X2_ on the *yz* plane and the *xy* plane in the initial state. Follow the above steps to transform the mid wing 2 and the outer wing 2, and then perform the return operation, thus the dynamic matrix of the aircraft can be updated to:

23 $${\varvec{M}}_{{{\text{a3}}}}^{{}} \left( {m_{{{\text{a3}}}} \left( t \right)} \right) = \left[ {{\varvec{M}}_{{{\text{r1}}}}^{yz} \left( {m_{{{\text{r}}1}} \left( t \right)} \right),{\varvec{M}}_{{{\text{r2}}}}^{yz} \left( {m_{{{\text{r2}}}} \left( t \right)} \right),{\varvec{M}}_{{{\text{w1}}}}^{yx} \left( {m_{{{\text{w}}1}} \left( t \right)} \right),{\varvec{M}}_{{{\text{w1}}}}^{yx} \left( {m_{{{\text{w}}2}} \left( t \right)} \right),{\varvec{M}}_{{\text{f}}}^{{}} \left( {m_{{\text{f}}} \left( {t{ = }0} \right)} \right)} \right]$$

where *m*_a3_ is the aircraft model in Z3 folding mode, ***M***_a3_ is the grid coordinate matrix of *m*_a3_. The irradiation area could be extracted as:

24 $$S_{{{\text{a3}}}} \left( t \right) \Leftarrow {\varvec{M}}_{{{\text{a3}}}}^{{}} \left( {m_{{{\text{a3}}}} \left( t \right)} \right)$$

where *S*_a3_ represents the irradiation area on the surface of *m*_a3_.

For the Z4 folding mode, the outer wing 1 translates with the mid wing 1 while also deflecting downwards:

25 $${\varvec{M}}_{{{\text{r1}}}}^{y} \left( {m_{{{\text{r1}}}} \left( t \right)} \right) = {\varvec{M}}_{{\text{r1}}} \left( {y\left( {m_{{{\text{r1}}}} \left( {t = 0} \right)} \right) - Y^{\prime}_{{{\text{r1}}}} \left( t \right)} \right)$$

26 $${\varvec{M}}_{{{\text{r1}}}}^{z} \left( {m_{{{\text{r1}}}} \left( t \right)} \right) = {\varvec{M}}_{{{\text{r}}1}}^{y} \left( {z\left( {m_{{{\text{r1}}}} \left( t \right)} \right) + Z^{\prime}_{{{\text{r1}}}} \left( t \right)} \right)$$

27 $${\varvec{M}}_{{{\text{r1}}}}^{x} \left( {m_{{{\text{r1}}}} \left( t \right)} \right) = \left[ {\begin{array}{*{20}c} 1 & 0 & 0 \\ 0 & {\cos A_{{{\text{r1}}}} \left( t \right)} & { - \sin A_{{{\text{r1}}}} \left( t \right)} \\ 0 & {\sin A_{{{\text{r1}}}} \left( t \right)} & {\cos A_{{{\text{r1}}}} \left( t \right)} \\ \end{array} } \right] \cdot {\varvec{M}}_{{{\text{r1}}}}^{z} \left( {m_{{{\text{r1}}}} \left( t \right)} \right)$$

The relevant change parameters are updated as follows:

28 $$Y^{\prime}_{{{\text{r1}}}} \left( t \right){ = }Y_{{{\text{m1}}}} + R_{0} \cos \left( {A_{{{\text{m1}}}} \left( t \right) - A_{0} } \right)$$

29 $$Z^{\prime}_{{{\text{r1}}}} \left( t \right){ = }Z_{{{\text{m1}}}} + R_{0} \sin \left( {A_{{{\text{m1}}}} \left( t \right) - A_{0} } \right)$$

30 $$A_{{{\text{r}} 1}} \left( t \right) = \arcsin \frac{{Z_{{{\text{m}} 1}} + R_{0} \sin \left( {A_{{{\text{m}} 1}} \left( t \right) - A_{0} } \right) + h_{0} }}{{R_{1} }}$$

where *h*_0_ is airfoil thickness at the root of outer wing 1, *R*_1_ is the length of outer wing 1. The matrix of the aircraft and the illuminated area can be updated to:

31 $${\varvec{M}}_{{{\text{a4}}}}^{{}} \left( {m_{{{\text{a4}}}} \left( t \right)} \right) = \left[ {{\varvec{M}}_{{{\text{r1}}}}^{xy} \left( {m_{{{\text{r}}1}} \left( t \right)} \right),{\varvec{M}}_{{{\text{r2}}}}^{xy} \left( {m_{{{\text{r2}}}} \left( t \right)} \right),{\varvec{M}}_{{{\text{w1}}}}^{yx} \left( {m_{{{\text{w}}1}} \left( t \right)} \right),{\varvec{M}}_{{{\text{w1}}}}^{yx} \left( {m_{{{\text{w}}2}} \left( t \right)} \right),{\varvec{M}}_{{\text{f}}}^{{}} \left( {m_{{\text{f}}} \left( {t{ = }0} \right)} \right)} \right]$$

32 $$S_{{{\text{a4}}}} \left( t \right) \Leftarrow {\varvec{M}}_{{{\text{a4}}}}^{{}} \left( {m_{{{\text{a4}}}} \left( t \right)} \right)$$

where *m*_a4_ is the aircraft model in Z4 folding mode, ***M***_a4_ is the grid coordinate matrix of *m*_a4_, where *S*_a4_ represents the irradiation area on the surface of *m*_a4_.

### Electromagnetic scattering calculation

There are large areas of curved surfaces on the fuselage and various parts of the wings, then PO method is used to solve the scattering contribution of the facets, where the scattered electric field in the far field can be described as:

33 $$E_{\text{s}}^{{\left( {m_{\text{a}} \left( t \right)} \right)}} = \frac{{\text{e}^{{ - {\text{j}}kr}} }}{{4\uppi r}}\left( { - {\text{j}}\omega \mu } \right)\int\limits_{S\left( t \right)} {{\varvec{J}}_{\text{s}}{\text{e}}^{{ -{\text{j}}k{\varvec{s}} \cdot \user2{r^{\prime}}}}{\text{d}}S} +{\text{j}}k{\varvec{s}}\frac{1}{\varepsilon }\int\limits_{S\left( t \right)} {\rho_{\text{s}}{\text{e}}^{{ -{\text{j}}k{\varvec{s}} \cdot \user2{r^{\prime}}}}{\text{d}}S} \left| {\begin{array}{*{20}c} {} \\ {\forall S\left( t \right) \in \left\{ {S_{{{\text{a}} 1}} \left( t \right),S_{{{\text{a}} 2}} \left( t \right),S_{{{\text{a}} 3}} \left( t \right),S_{a4} \left( t \right)} \right\}} \\ \end{array} } \right.$$

where *k* is the wave number in free space, *μ* is the magnetic permeability, *ω* is the angular frequency, ***J***_s_ is the surface current, ***s*** means radiation direction,* r* is the field point, ***r***′ is the source point coordinate vector, *ε* is the dielectric constant, *ρ*_s_ is the charge density, d*S* is the integral facet.

For plane waves, the RCS contributed by the facets can be calculated as:

34 $$\sqrt {\sigma_{\text{F}} \left( t \right)} = \mathop {\lim }\limits_{R \to \infty } \sqrt {4\uppi R^{2} } \frac{{{\varvec{e}}_{\text{s}} \cdot {\varvec{E}}_{\text{s}}^{{\left( {m_{\text{a}} \left( t \right)} \right)}} }}{{\left| {{\varvec{E}}_{\text{i}} } \right|}}$$

where *σ* is the radar cross-section, subscript F represents the facet contribution, *R* is the distance between the field point and the source point, *E*_i_ is the electric field intensity at the point of incidence, ***e***_s_ is the direction of the scattered electric field. Further transform the RCS calculation formula:

35 $$\sqrt {\sigma_{\text{F}} \left( t \right)} = \sqrt {4\uppi R^{2} } \frac{1}{{\left| {{\varvec{E}}_{\text{i}} } \right|}}\left( { -{\text{j}}\omega \mu \frac{{\text{e}^{{ -{\text{j}}kr}} }}{{4\uppi r}}\int\limits_{S\left( t \right)} {{\varvec{e}}_{\text{s}} \cdot {\varvec{J}}_{\text{s}}{\text{e}}^{{ -{\text{j}}k{\varvec{s}} \cdot \user2{r^{\prime}}}}{\text{d}}S} } \right)$$

Noting that the surface current could be expressed as:

36 $${\varvec{J}}_{\text{s}} = \left\{ {\begin{array}{*{20}c} {2{\text{n }} \times {\text{H}}_{\text{i}} } \\ {0} \\ \end{array} } \right.\begin{array}{*{20}c} {} & {\begin{array}{*{20}c} {\begin{array}{*{20}c} , & {Z_{\text{I}} } \\ \end{array} } \\ {\begin{array}{*{20}c} , & {Z_{\text{D}} } \\ \end{array} } \\ \end{array} } \\ \end{array}$$

where ***n*** is the normal vector outside the surface element, *Z*_I_ is the illuminated area, *Z*_D_ is the unilluminated area, ***H***_i_ represents the magnetic field intensity of the incident point. Considering the relationship between electric field and magnetic field, the facet RCS can be obtained as:

37 $$\sqrt {\sigma_{\text{F}} \left( t \right)} = \frac{{\text{j}k}}{{\sqrt\uppi }}{\varvec{n}} \cdot \left( {{\varvec{e}}_{\text{s}} \times {\varvec{h}}_{\text{i}} } \right)\text{e}^{{\text{j}k{\varvec{w}} \cdot {\varvec{r}}_{\text{0}} }} I\left( t \right)$$

where ***r***_0_ is the coordinate vector of the reference point on the integral surface element, ***h***_i_ is the direction of the incident magnetic field, ***i*** is the unit vector of the incident wave, *I*(*t*) is an integral expression, which can be obtained according to the following calculation when the triangular facets are used:

38 $${\text{w}} = {\text{s}} - {\text{i}}$$

39 $$I\left( t \right) = \left\{ {\begin{array}{*{20}l} {\frac{{\text{e}^{{\text{j}k{\varvec{w}} \cdot {\varvec{r}}_{\text{m}} \left( t \right)}} }}{{\text{j}k\left| {\varvec{p}} \right|^{2} }}\sum\limits_{m = 1}^{3} {{\varvec{p}} \cdot {\varvec{L}}_{\text{m}}{\text{sinc}}\left( {\frac{{k{\varvec{w}} \cdot {\varvec{L}}_{\text{m}} }}{2}} \right),} } \hfill & {\left| {\varvec{p}} \right| \ne 0} \hfill \\ {A_{\text{f}} ,} \hfill & {\left| {\varvec{p}} \right| = 0} \hfill \\ \end{array} } \right.$$

where ***L***_m_ is the vector of the *m*-th edge on the facet, *A*_f_ is the area of the integral facet, noting that ***p*** is a defined vector cross product:

40 $${\text{sinc}}\left( x \right) = \sin x/x$$

41 $${\text{p}} = {\text{n}} \times {\text{w}}$$

PTD is used to solve the edge diffraction contribution of the target, then the total RCS could be obtained as:

42 $$\sigma \left( t \right) = \left| {\sum\limits_{i = 1}^{{N_{F} (t)}} {\left( {\sqrt {\sigma_{\text{F}} (t)} } \right)} \begin{array}{*{20}c} {} \\ i \\ \end{array} + \sum\limits_{j = 1}^{{N_{E} (t)}} {\left( {\sqrt {\sigma_{\text{E}} (t)} } \right)\begin{array}{*{20}c} {} \\ j \\ \end{array} } } \right|^{2} ,\begin{array}{*{20}c} {} & {t \in \left[ {0,T_{{\text{ob}}} } \right]} \\ \end{array}$$

where *σ*_E_ is the radar cross-section with the edge contribution. *N*_F_ is the number of facets, *N*_E_ is the number of edges, and *T*_obs_ is the recommended length of time for observation, in order to ensure that the predetermined deflection action is completed. For more descriptions of PO + PTD, please refer to the literature^18,21^.

To visually analyze and judge the scattering effect of the target surface, a custom surface scattering intensity could be written as follows:

43 $$I_{{\text{ss}}} \left( i \right) = K_{{\text{cd}}} \left( {B_{cm} - B_{cn} } \right)\frac{{\sigma_{\text{f}} \left( i \right) - \sigma_{{\text{fn}}} }}{{\sigma_{{\text{fm}}} - \sigma_{{\text{fn}}} }} + K_{{\text{cd}}} \cdot B_{cn}$$

where *I*_ss_(*i*) is custom surface scattering intensity, *σ*_f_ represents the facet RCS under the current condition, the *i* in brackets is the facet number, *K*_cd_ is the color depth control factor, *σ*_fn_ is the minimum of the facet RCS, *σ*_fm_ is the maximum of the facet RCS, *B*_cm_ and *B*_cn_ represent the upper and lower boundaries of the current color window, respectively. For more details on dynamic RCS, refer to reference^26^.

### Method verification

**Figure 2:**
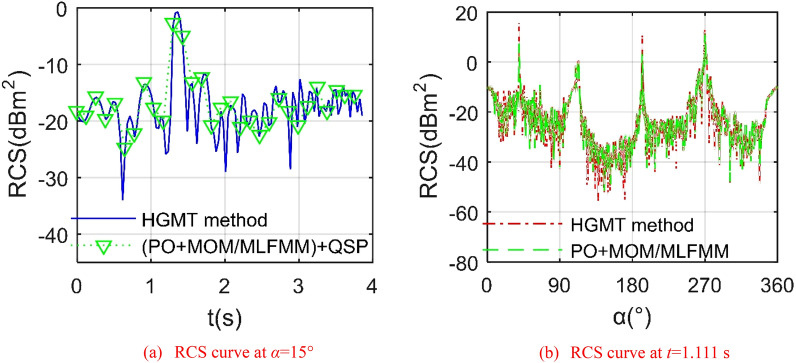
The Validation of HGMT on the outer wing 1, *f*_RH_ = 6 GHz, *β* = 3°, *ω*_r1_ = 0.3142 rad/s.

The Validation of HGMT method is presented in Fig. 2, where *f*_RH_ represents to radar wave frequency and horizontal polarization, *ω*_r1_ is deflection angular velocity of outer wing 1. For the RCS ~ *t* curves, PO + MOM (method of moment)/MLFMM (multi-level fast multipole method) in FEKO (FEldberechnung bei Korpern mit beliebiger Oberflache) is used to calculate the RCS of the target, where QSP is used to discretize the deflection state of outer wing 1. The mean RCS of HGMT curve is − 13.59 dBm^2^, while that of discrete points obtained by FEKO is − 13.24 dBm^2^. At *t* = 1.944 s, the outer wing 1 deflected down by 34.992°, where the RCS of the HGMT curve is − 18.32 dBm^2^, that of the other curve is − 17.66 dBm^2^. It could be found that the HGMT curve passes through the FEKO discrete points well, and different calculation methods lead to the local and mean errors of RCS. In addition to the errors caused by different calculation methods, there are also differences between the unstructured mesh generation used in this paper and the mesh generation used in FEKO. In order to obtain the calculation model at different times, QSP needs to pay a lot of work and operation steps. HGMT can continuously and conveniently handle RCS changes caused by wing deflection. These results show that HGMT is feasible and accurate, and is used to calculate the electromagnetic scattering characteristics of the wing during continuous deflection.

For the RCS ~ *α* curves, the downward deflection angle of the outer wing 1 is equal to 19.998°. It can be found that the two RCS curves are roughly similar, including shape, peak position and number of peaks. The mean RCS of the HGMT curve is − 8.39 dBm^2^, that of the other is − 8.54 dBm^2^. When the azimuth angle is equal to 270°, the RCS of HGMT is 13.03 dBm^2^, and the RCS of FEKO is 12.33 dBm^2^. For RCS ~ *α* curve and RCS ~ *β* curve, the existing technologies including MOM and PO methods are feasible and widely used. Other obvious differences are reflected in the number of minima and individual maxima, because different calculation methods and the difference in the number of curve base points will bring errors to the indicator. The current calculation state is only an instantaneous state, and the different folding modes of the variant aircraft reflect complex dynamic changes, which is obviously inconvenient to deal with by conventional methods. These results show that HGMT is feasible and accurate to deal with the electromagnetic scattering characteristics of the target in transient state and dynamic folding.

## Model

**Figure 3:**
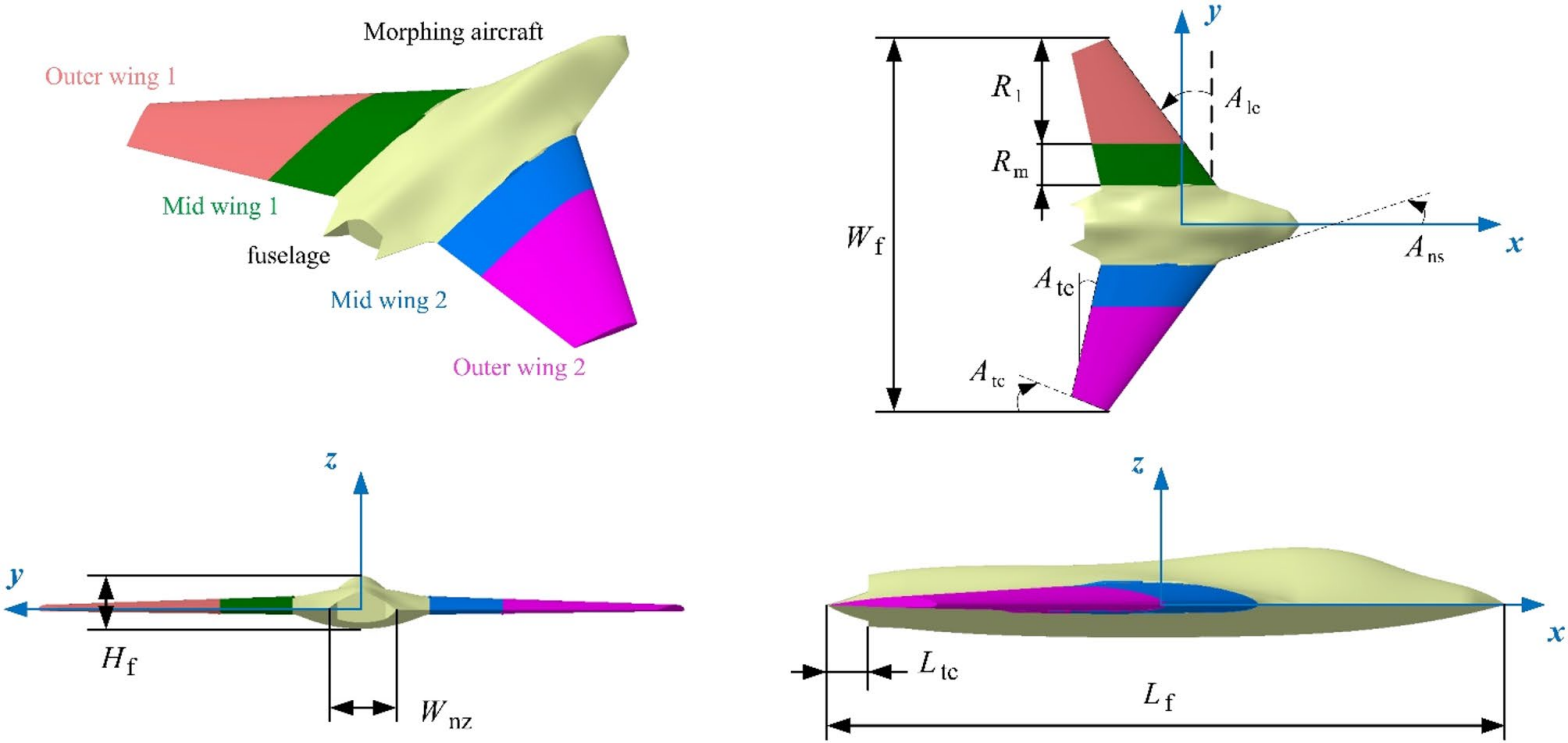
Aircraft model and main parameter distribution.

The model of the aircraft is built as shown in Fig. 3, where *R*_m_ is the length of mid wing 1, *L*_f_ is the length of the aircraft fuselage, *A*_le_ is the sweep angle of the leading edge of the wing, *A*_te_ is the sweep angle of the trailing edge of the wing, *W*_f_ is the wingspan of the aircraft, *A*_tc_ is the cutting angle of the wing tip, *H*_f_ is the height of the aircraft fuselage, *A*_ns_ is the angle of the sideline of the nose, *W*_nz_ is the outer width of the nozzle, *L*_tc_ is the cut length of the tail of the fuselage. According to the actual needs, the actual folding angle of the four modes can be larger within the allowable range. When the rotation axis of the folding action is set at another appropriate position, the folding mode will change.

The main size of the aircraft model is presented in Table 1, where this aircraft uses a symmetrical design, which makes the outer wing 1 and outer wing 2 symmetrically distributed about the *xz* plane, and the mid wing 1 and mid wing 2 are symmetrical about the *xz* plane. The leading edge and trailing edge of the wing are designed with a swept back, and the wing tip has been cut off.

**Table 1:** The main size distribution of the aircraft model.

Parameter	*L*_f_ (m)	*W*_f_ (m)	*A*_le_ (°)	*H*_f_ (m)	*A*_te_ (°)	*A*_tc_ (°)
Value	9.911	16	36.713	1.312	12.515	22.35

Parameter	*L*_tc_ (m)	*R*_1_ (m)	*R*_m_ (m)	*A*_ns_ (°)	*h*_0_ (m)	*W*_nz_ (m)
Value	0.611	4.5	1.8	17.463	0.365	1.728

**Figure 4:**
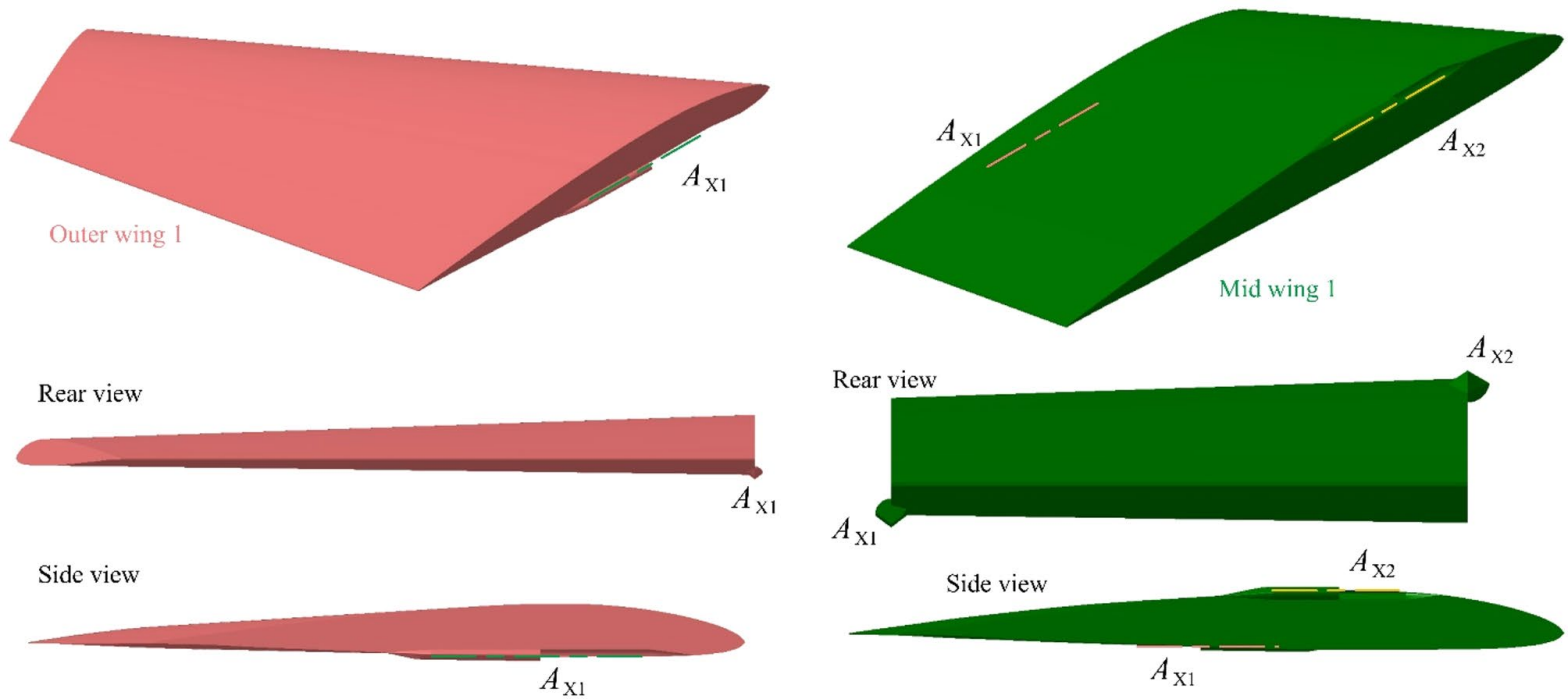
Detail display of outer wing 1 and mid wing 1.

The details of the outer wing 1 are shown in Fig. 4, where the outer wing 1 and mid wing 1 are combined by *A*_X1_, and mid wing 1 and fuselage are combined by *A*_X2_. For the rotation axis *A*_X1_ and *A*_X2_ of the wing, the fairing is narrow and sharp as a whole, and the cross section is a combination of partial arcs and V shapes. Note that *A*_X1_ is set below the wing and *A*_X2_ is set above the wing. The V shape is placed on the surface of the wing and the arc is placed inside the plane.

**Figure 5:**
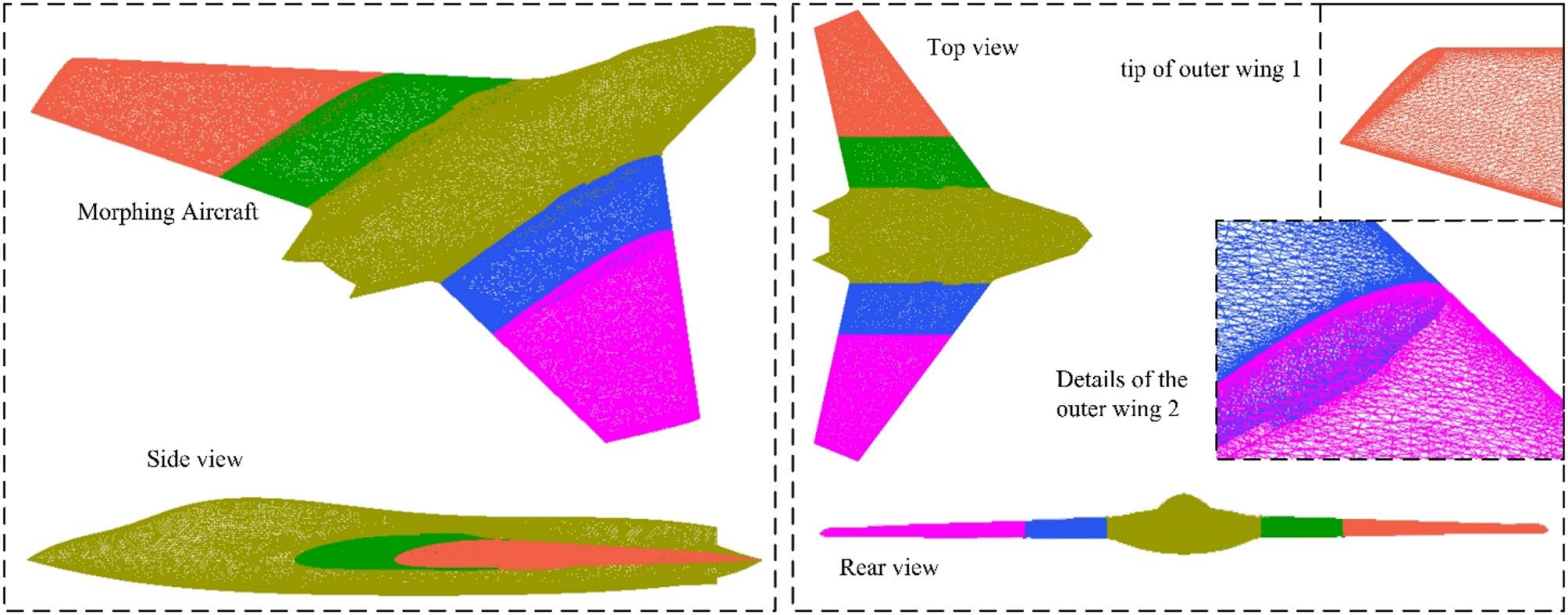
Meshing of the surface of each part of the aircraft.

The surface of the fuselage and each section of the wing is processed with high-precision unstructured grid technology as shown in Fig. 5, where the outer wing and the middle wing are not deflected in the initial state. For the outer wing, the leading edge, trailing edge, wing tip, and rotating shaft of the wing are areas of smaller size, where mesh density increase technology needs to be applied.

The mesh size distribution on the aircraft surface is provided in Table 2, where mesh density increase technology is widely used in curved surfaces with large changes in curvature or small area, including nose curve, fuselage edge, nozzle, wing leading edge, wing tip, wing shaft and wing trailing edge. Note that each outer wing has one shaft, and each mid wing has two shafts.

**Table 2:** The mesh size distribution on the surface of each part of the aircraft.

Area	Max size (mm)	Area	Max size (mm)
Global minimum size	1	Outer wing trailing edge	2
Outer wing leading edge	3	Wingtip curve	3
Mid wing leading edge	5	Mid wing trailing edge	5
Wing shaft surface	6	Nose curve	10
Edge of the fuselage	15	Tail line of fuselage	15
Wing end face	20	Outer wing surface	25
Mid wing surface	30	Fuselage surface	50

## Results and discussion

**Figure 6:**
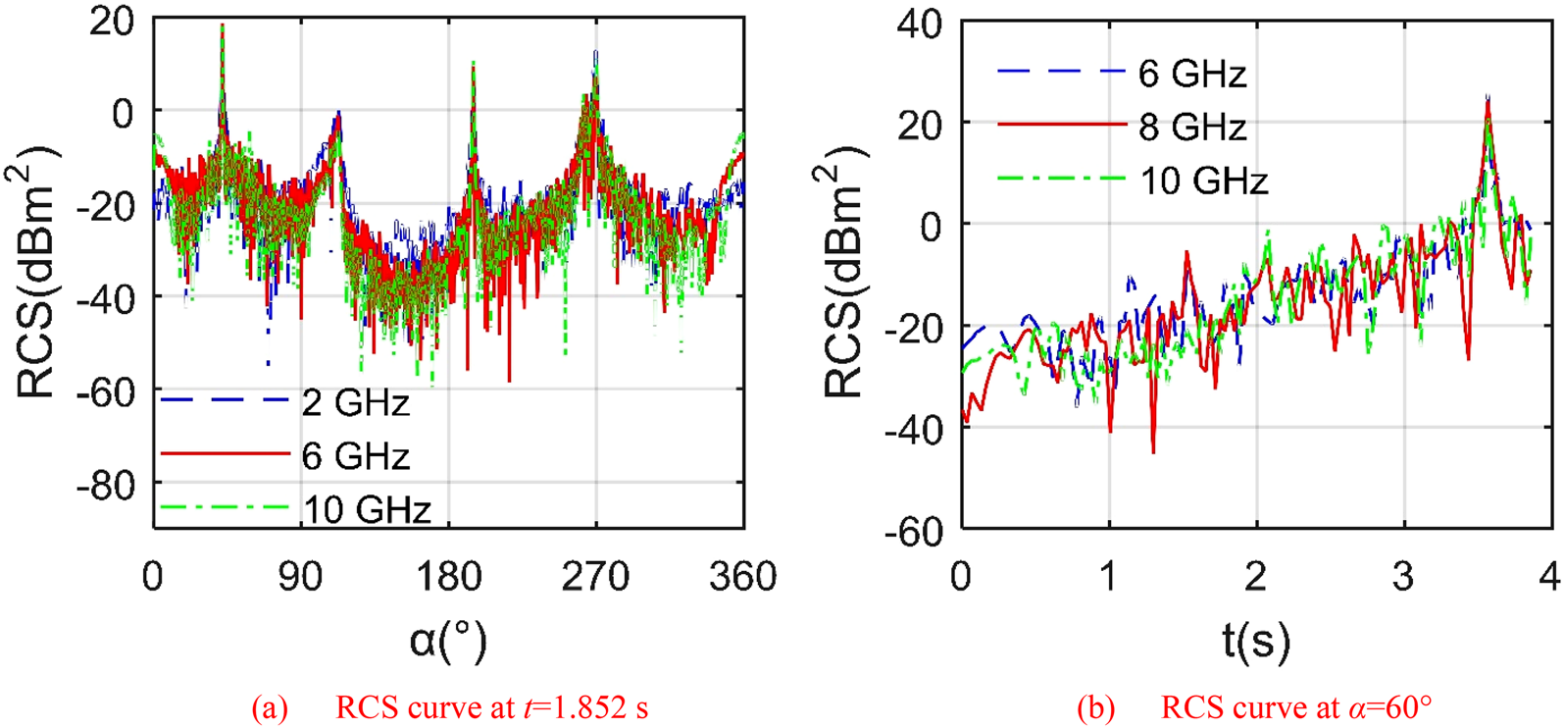
RCS of the outer wing 1 under different radar wave frequencies, *β* = 0°, *ω*_r1_ = 0.3142 rad/s.

Figure 6 presents that the increase of radar wave frequency brings obvious changes to the RCS ~ *α* and RCS ~ *t* curves of outer wing 1. For the RCS ~ *α* curve, the outer wing 1 deflected downward by 33.336°, where the mean value of the RCS curve under 6 GHz is equal to − 8.9423 dBm^2^, that under 10 GHz is equal to − 8.2669 dBm^2^. It can be seen that the overall trend and peak value of these 3 RCS curves are similar, and there are many differences in local fluctuations. For the RCS ~ *t* curve, the outer wing 1 continuously deflects downwards from a horizontal position. There are obvious differences in the mean, local fluctuation and peak value of these three RCS curves, where the peak of the RCS curve under 6 GHz is equal to 25.7755 dBm^2^, that under 8 GHz is equal to 24.1351 dBm^2^ as shown in Table 3. For the RCS curve at 10 GHz, it can be seen that the RCS value increases with the increase of the deflection angle, and is accompanied by many violent jumps, because under the current observation conditions, the angle between the illumination area on the upper surface of the outer wing 1 and the radar wave gradually increases as the outer wing 1 continues to deflect downward. In this process, the strong scattering source gradually shifts from the front surface to the upper surface. At an azimuth angle of 60°, the average RCS index of the outer wing 1 generally decreases with the increase of the radar wave frequency. These results indicate that the influence of the wing folding action on its radar stealth characteristics cannot be ignored.

**Table 3:** RCS indicator of outer wing 1, *α* = 60°, *β* = 0°, *ω*_r1_ = 0.3142 rad/s, *t* = 0 ~ 3.857 s.

*f*_RH_ (GHz)	2	4	6	8	10	12
Mean (dBm^2^)	5.5538	6.6450	5.4119	4.4112	2.0405	-0.4535
Peak (dBm^2^)	22.8069	25.8010	25.7755	24.1351	21.4936	17.5069

### Analysis of Z1 mode

**Figure 7:**
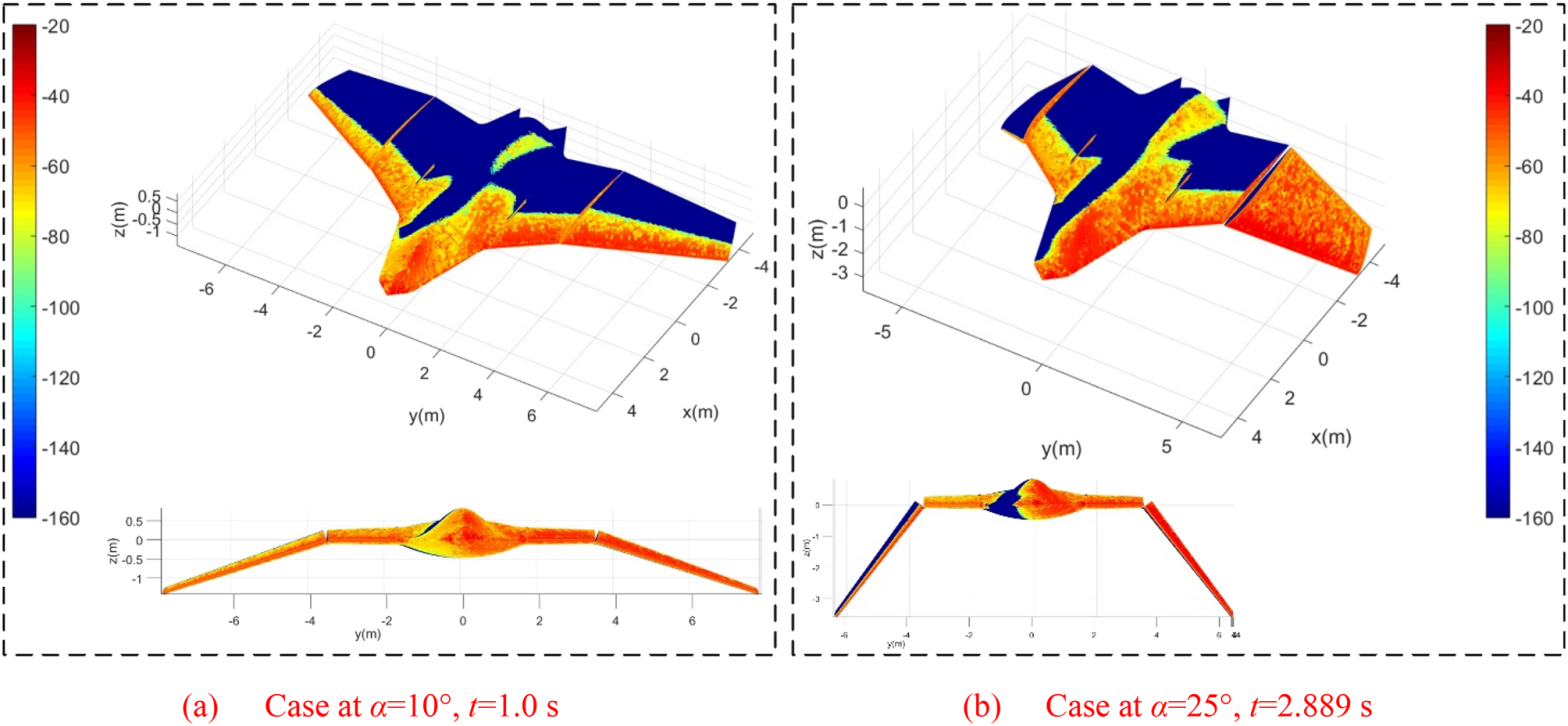
Surface scattering characteristics of aircraft in Z1 mode, *β* = 0°, *f*_RH_ = 6 GHz, *ω*_r1_ = *ω*_r2_ = 0.3142 rad/s, *A*_m1_ = *A*_m2_ = 0°, RCS unit: dBm^2^.

Figure 7 supports that under current observation conditions, the Z1 mode will significantly increase the strong scattering sources on the aircraft surface. For the case of *α* = 10° and *t* = 1.0 s, the outer wing deflects downward by 18°, where the mid wing and fuselage are kept level. It can be found that the nose, the top of the cockpit, the fusion of the wing fuselage and the leading edge of the wing have more red distribution. There is a small amount of yellow-green at the rear of the fuselage. When *α* = 25° and *t* = 2.889 s, the increase in azimuth brings more red to the fusion of the nose, cockpit, and wing fuselage, while has less impact on the strong scattering source of the mid wing. At this time, the outer wing deflects 52.002° downward, where the scattering enhancement effect brought by the folding action to the outer wing is very obvious, because both the upper surface of the outer wing 1 and the lower surface of the outer wing 2 are completely illuminated by radar waves. In addition, the gap between the outer wing and the middle wing is increased, and the scattering intensity of the end face of the mid wing is also increased. These results show that the HGMT method is feasible and intuitive to describe the electromagnetic scattering characteristics of the variant aircraft when the wings are folded.

**Figure 8:**
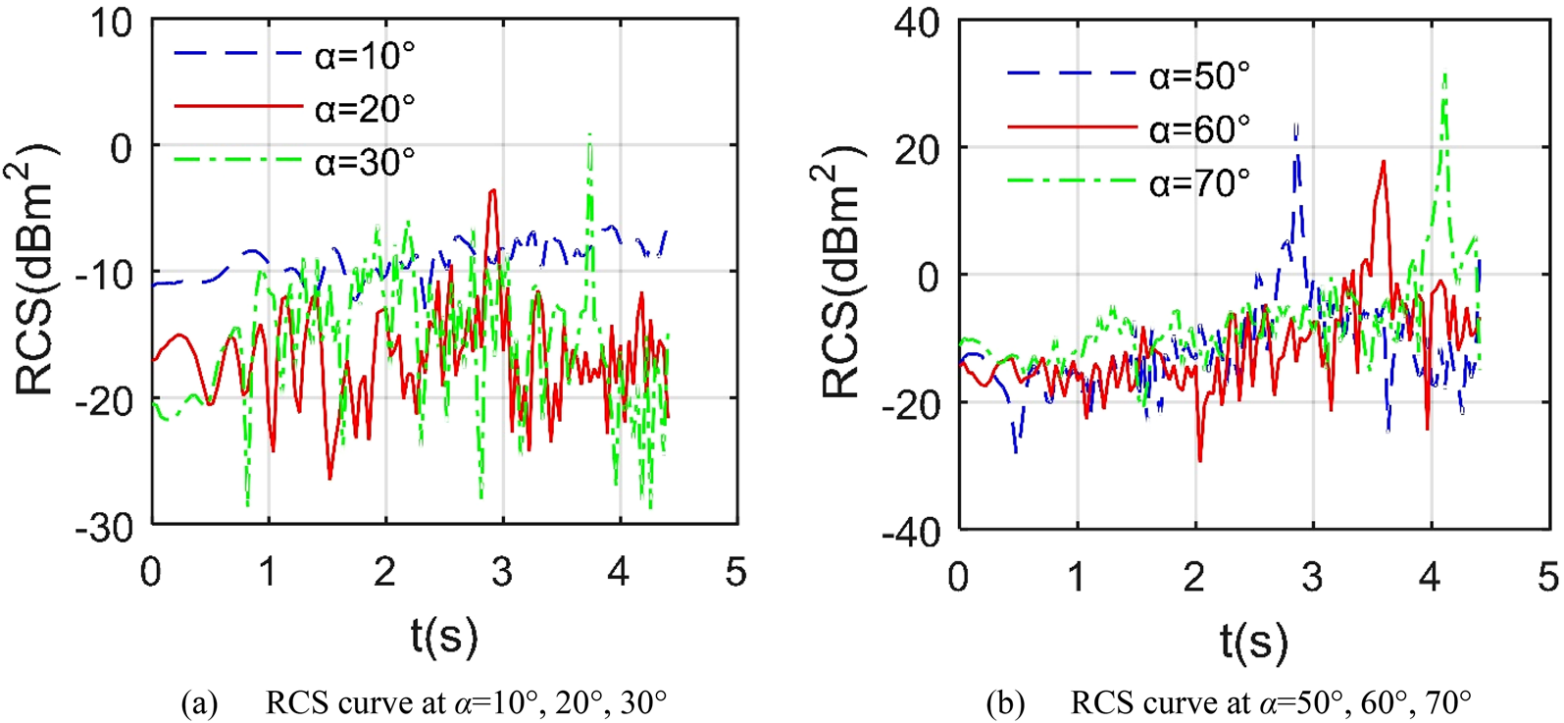
The influence of different azimuths on dynamic RCS of the aircraft in Z1 mode, *β* = 0°, *f*_RH_ = 6 GHz, *ω*_r1_ = *ω*_r2_ = 0.3142 rad/s, *A*_m1_ = *A*_m2_ = 0°.

Figure 8 indicates that the dynamic RCS curve of the aircraft under different azimuth angles is quite different, including the fluctuation range, peak size, peak number and change trend. For the RCS curve at *α* = 10°, the curve has small fluctuations between − 12.97 and − 6.186 dBm^2^, because the strong scattering sources on the aircraft surface are mainly concentrated on the fuselage head and the leading edge of the wing, and the deflection of the outer wing has little effect on the illumination area. When *α* = 20°, the fluctuation range of the RCS curve has been greatly increased, ranging from − 26.53 to − 3.521 dBm^2^, which is mainly due to the scattering effect of the front edge of the outer wing and its nearby curved surface. This increased volatility is also reflected in the RCS curve of *α* = 30°, where the maximum value has reached 1.093 dBm^2^. For the case of *α* = 50°, the fluctuating curve has an obvious large peak, which is 23.85 dBm^2^ at 2.852 s, because the incident wave at this time is incident sideways, the upper surface of the outer wing 1 becomes a dynamic scattering source. This prominent peak feature also exists on the curves of *α* = 60° and *α* = 70°, where the maximum values are 17.88 dBm^2^ and 32.32 dBm^2^ respectively. These results indicate that the azimuth angle has a greater impact on the dynamic RCS of the aircraft in the Z1 mode, and the RCS curve near the side fluctuates more obviously than the head.

**Figure 9:**
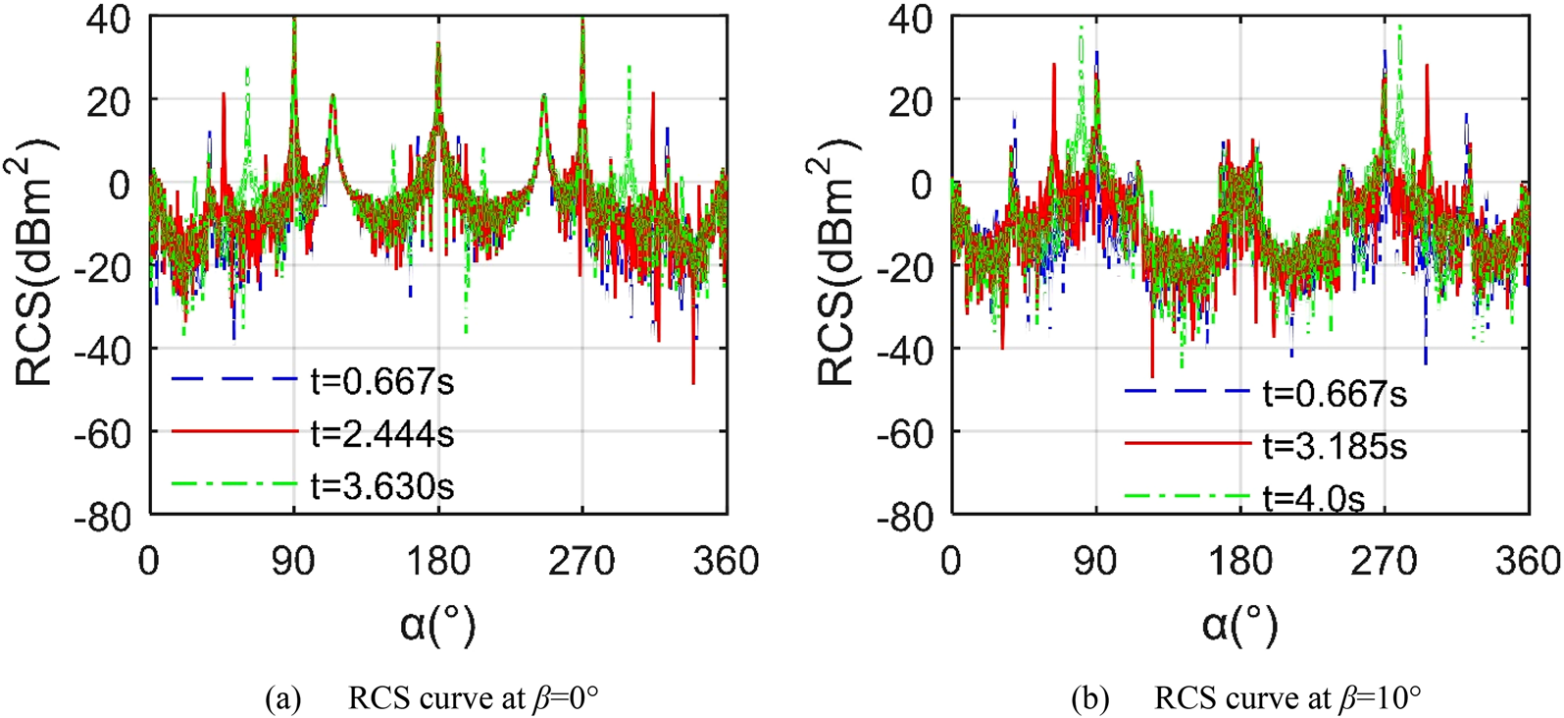
RCS of the aircraft in Z1 mode, *f*_RH_ = 6 GHz, *ω*_r1_ = *ω*_r2_ = 0.3142 rad/s, *A*_m1_ = *A*_m2_ = 0°.

Figure 9 reveals that at different instantaneous moments, the RCS ~ *α* curve of the aircraft will show obvious differences, including the mean value and the local peak value. For the RCS curve at *t* = 0.667 s, the two largest peaks appear in the lateral direction, 39.13 dBm^2^ at *α* = 90.25° and 39.21 dBm^2^ at *α* = 270.3° respectively, where the mean RCS of the curve is equal to 15.553 dBm^2^. When *t* = 2.444 s, the RCS curve has a peak of 21.58 dBm^2^ at *α* = 46°, because the outer wing 1 deflected 43.992° downwards, the end face of the mid-section wing 1 is exposed, and the upper surface of the outer wing 1 is also not conducive to deflecting radar waves to a non-threatening direction. For the case of *t* = 3.630 s, the mean index of the RCS curve increased to 16.003 dBm^2^, while the change of the maximum peak is not obvious. When *β* = 10°, the peak and average levels of the RCS curve with *t* = 4.0 s are significantly higher than the other two, where the mean of the RCS curve at *t* = 0.667 s is 7.373 dBm^2^, that at *t* = 3.185 s is 6.227 dBm^2^ as shown in Table 4. It is worth noting that increasing the elevation angle from 0° to 10° significantly reduces the average index of the RCS curve at *t* = 0.667 s. These results show that although the aircraft in Z1 mode only deflects the outer wing, this folding action will bring a non-negligible change to the stealth characteristics of the aircraft.

**Table 4:** RCS indicator of the aircraft in Z1 mode, *α* = 0° ~ 360°, *f*_RH_ = 6 GHz, *ω*_r1_ = *ω*_r2_ = 0.3142 rad/s, *A*_m1_ = *A*_m2_ = 0°.

*t* (s)	*β* = 0°			*β* = 10°		
	0.667	2.444	3.630	0.667	3.185	4.0
Mean (dBm^2^)	15.5527	15.7285	16.0027	7.3727	6.2272	12.8321
Peak (dBm^2^)	39.2094	39.7478	39.8298	32.1615	28.6215	37.9374

### Analysis of Z2 mode

**Figure 10:**
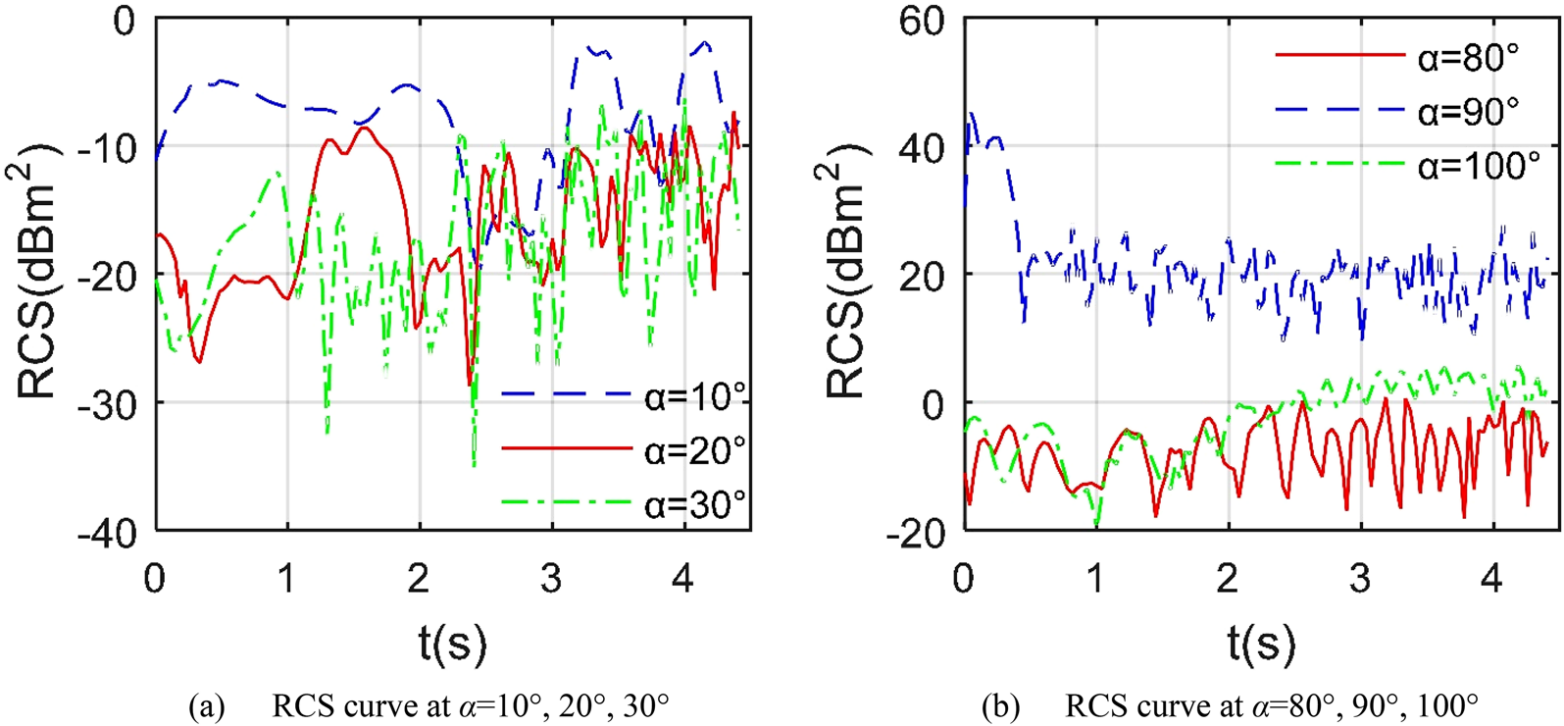
RCS of the aircraft under different azimuths, *β* = 0°, *f*_RH_ = 6 GHz, *ω*_m1_ = *ω*_m2_ = 0.1963 rad/s.

Figure 10 manifests that under the given head azimuth angle, the RCS curve in Z2 mode fluctuates more than in Z1 mode. When *α* = 10°, the minimum value of the RCS curve is as low as − 20.23 dBm^2^ at 2.444 s, and the maximum value is -1.956 dBm^2^ at 4.148 s as shown in Table 5. For the case of *α* = 20°, the minimum RCS is − 28.79 dBm^2^ while that of the RCS curve at *α* = 30° is − 35.16 dBm^2^. The main contribution of the increase in RCS fluctuations under heading azimuth is mainly from the dynamic deflection changes of the leading edge of the entire wing. Compared with the Z1 mode, the outer end wing and the middle wing in the Z2 mode deflect upward together. Consider the situation under lateral azimuth, the RCS curve when *α* = 80° fluctuates sharply between − 18.18 and 0.7757 dBm^2^, where the mean index is only equal to − 5.65 dBm^2^. For the RCS curve at *α* = 90°, it can be clearly observed that the overall level of this curve is higher than the other two, where the mean RCS of the curve at *α* = 90° is 30.7186 dBm^2^, that at *α* = 100° is -0.0831 dBm^2^, because when the radar wave is incident from the positive side direction, the side of the fuselage, the tail nozzle baffle and the cockpit curved surface will provide more strong scattering sources, while the wing tip surface, wing tip edge and illuminated area curved surface of the entire wing will bring dynamic changes to the aircraft RCS.

**Table 5:** RCS indicator of the aircraft, *β* = 0°, *f*_RH_ = 6 GHz, *ω*_m1_ = *ω*_m2_ = 0.1963 rad/s, *t* = 0 ~ 4.407 s.

*α* (°)	10	20	30	80	90	100
Mean (dBm^2^)	− 6.6604	− 13.5478	− 14.5690	− 5.6471	30.7186	− 0.0831
Peak (dBm^2^)	− 1.9564	− 7.3101	− 6.2955	0.7757	45.8095	5.6211

**Figure 11:**
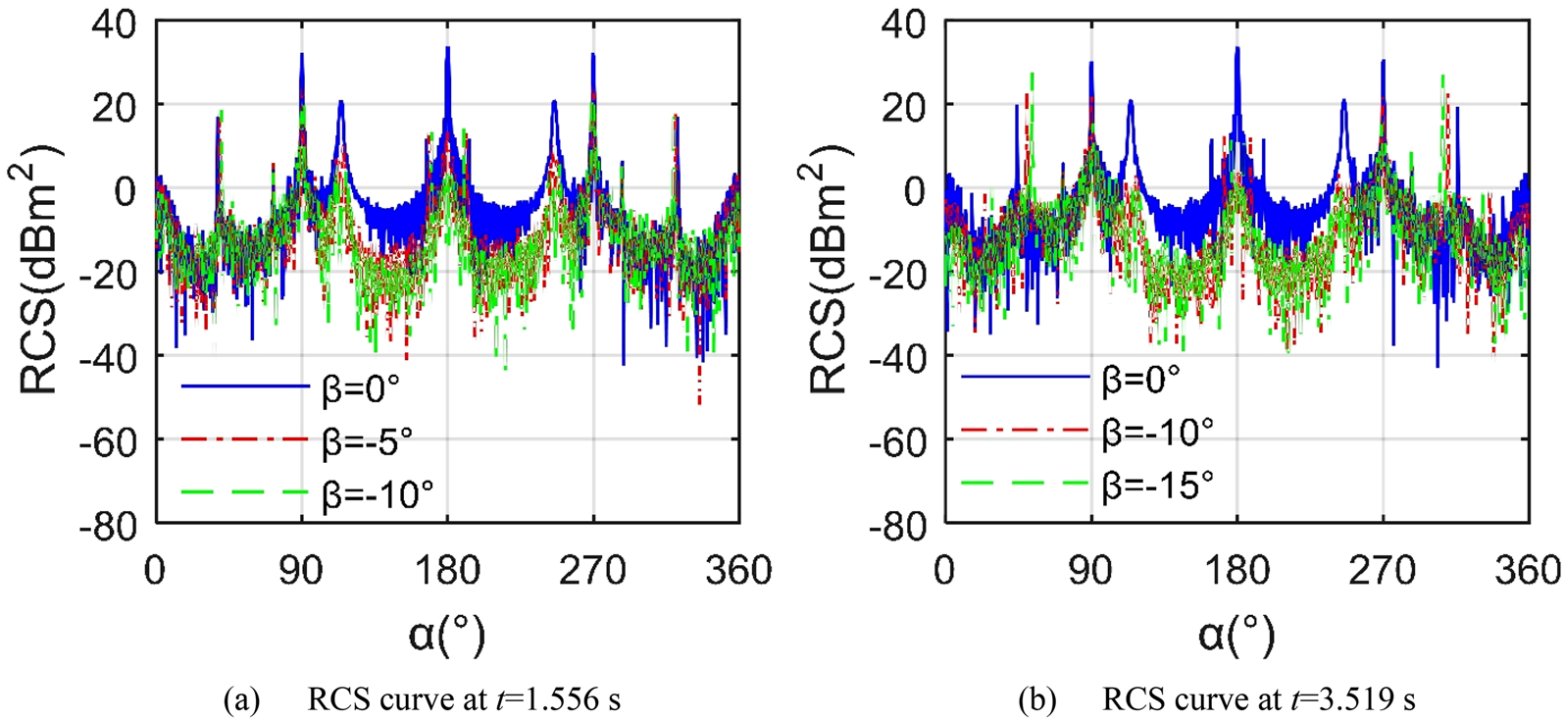
RCS of the aircraft under different elevation angle, *f*_RH_ = 6 GHz, *ω*_m1_ = *ω*_m2_ = 0.1963 rad/s.

Figure 11 shows that under the given observation conditions, the decrease of the elevation angle can significantly reduce the average level of the aircraft RCS curve. When *t* = 1.556 s, the RCS curve under *β* = 0° shows 3 large peaks, 32.24 dBm^2^ at *α* = 90.25°, 33.76 dBm^2^ at *α* = 179.8° and 32.34 dBm^2^ at *α* = 269.8°, where the tail end face of the fuselage and the trailing edge of the wing make the main contribution to the maximum tail peak. The average index of the RCS curve with *β* = − 5° dropped rapidly to 2.42 dBm^2^, while the average index of the RCS curve with *β* = − 10° was as low as 0.31 dBm^2^ as shown in Table 6, because the reduction of the elevation angle can effectively improve the distribution of strong scattering sources on the upper surface of the fuselage and near the nozzle. For the case of *t* = 3.519 s, the wing deflects upward by 39.579°, the average index of the RCS curve with *β* = − 15° is slightly higher than that with *β* = − 10° by 0.1045 dBm^2^. It is worth noting that when the elevation angle is equal to − 10°, the mean RCS of *t* = 1.556 s is higher than the index of *t* = 3.519 s. These results show that the Z2 mode has a profound impact on the aircraft RCS, including dynamic amplitude, average value and peak value.

**Table 6:** RCS indicator of the aircraft, *α* = 0° ~ 360°, *f*_RH_ = 6 GHz, *ω*_m1_ = *ω*_m2_ = 0.1963 rad/s.

*β* (°)	*t* = 1.556 s			*t* = 3.519 s		
	0	− 5	− 10	0	− 10	− 15
Mean (dBm^2^)	11.8451	2.4182	0.3109	11.5293	1.5218	1.6263
Peak (dBm^2^)	33.7636	24.9116	20.8882	33.7569	23.1870	27.7457

### Analysis of Z3 mode

**Figure 12:**
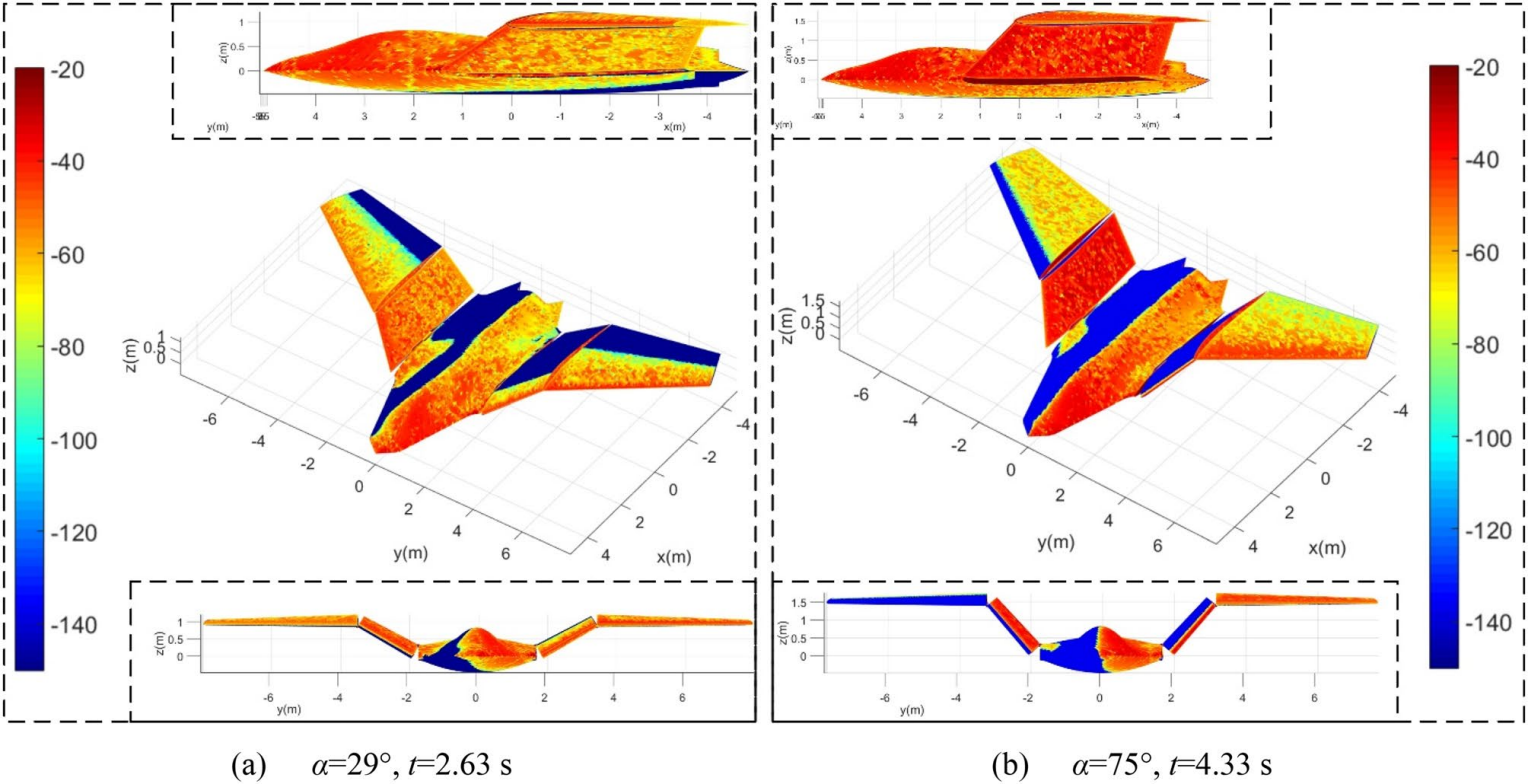
Surface scattering characteristics of aircraft in Z3 mode, *β* = 5°, *f*_RH_ = 6 GHz, *ω*_m1_ = *ω*_m2_ = 0.1963 rad/s, RCS unit: dBm^2^.

Figure 12 presents that in the Z3 folding mode, the radar waves incident from the side will cause more strong scattering sources on the surface of the aircraft than those from the head. When *α* = 29° and *t* = 2.63 s, the mid wing deflects upwards by 29.58°, and the outer wing remains level. The nose, the cockpit surface, the leading edge and outer end face of the mid wing 1, the leading edge of the outer wing 1, and the upper surface of the mid wing 2 all show red and orange red. The surfaces near the trailing edge of outer wing 1 and outer wing 2 both show low-intensity scattering areas. For the case of *α* = 75°, *t* = 4.33 s, the orange-red in the lighting area of the fuselage changed to red, and the original red was further deepened, which is mainly due to the increase in the azimuth angle of the incident radar wave. The red range of the lower surface of the mid wing 1 and the upper surface of the mid wing 2 is enlarged and deepened because of the combined effect of wing deflection and azimuth angle change. In addition, the upper surface of the outer wing 1 is all lit, while the leading edge of the outer wing 2 is transformed into a low scattering intensity area. The red color of the outer end face of the mid-section wing 1 is deepened, and the red color of the inner end face of the outer wing 2 turns to dark red. Due to the lateral offset of the azimuth angle, the orange and yellow colors in the low-scattering half of the fuselage are further reduced. These results indicate that although the outer wing remains level, the strong scattering source brought by the mid-section wing surface and the leading edge of the outer wing is still considerable under current observation conditions.

**Figure 13:**
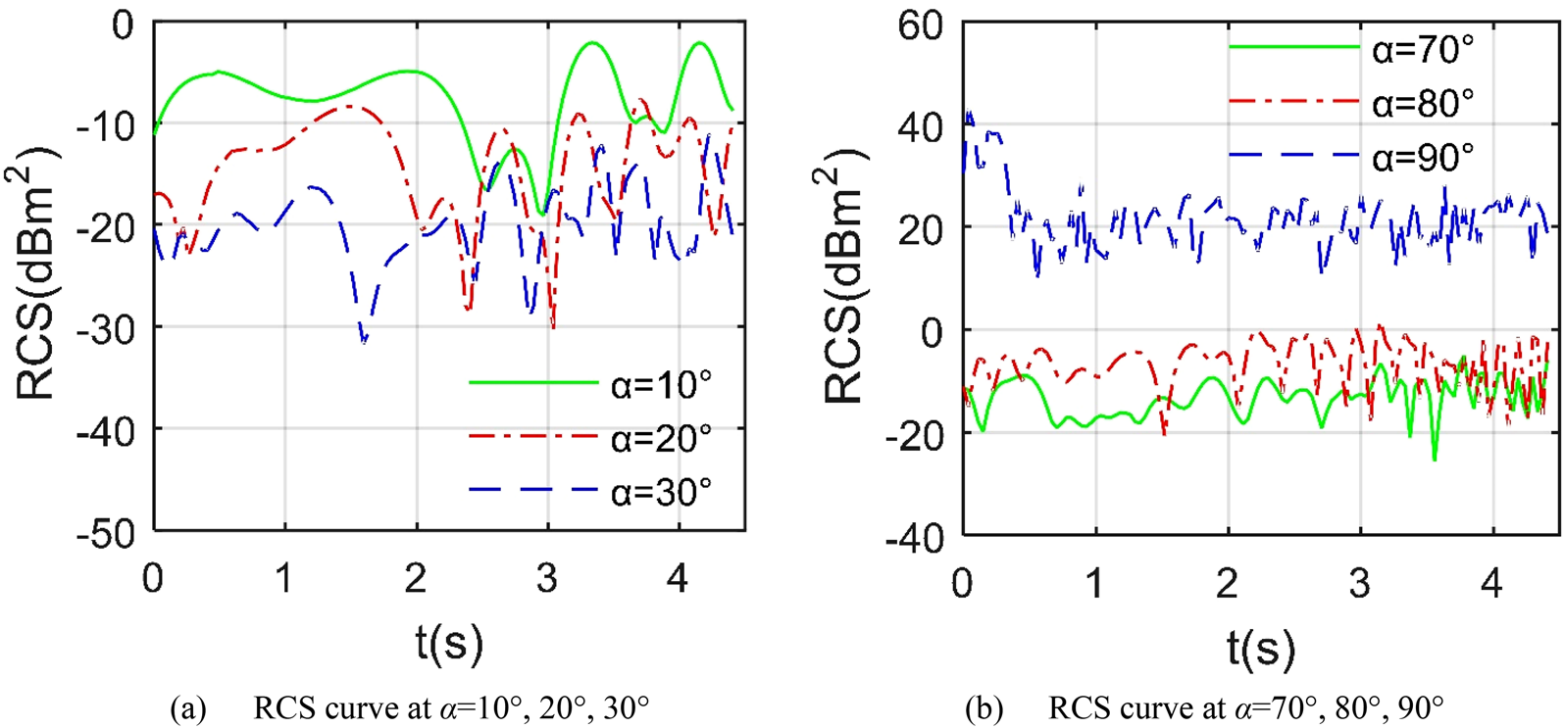
RCS of the aircraft in Z3 mode under various azimuths, *β* = 0°, *f*_RH_ = 6 GHz, *ω*_m1_ = *ω*_m2_ = 0.1963 rad/s.

Figure 13 provides that under the same azimuth, the dynamic RCS curve of the aircraft in Z3 mode is obviously different from the previous two modes. For the RCS curve at *α* = 10°, the minimum value is equal to -19.8 dBm^2^ which occurs at *t* = 2.963 s, where the mean RCS is − 6.5051 dBm^2^ as shown in Table 7. The fluctuation range of the RCS curve of *α* = 20°is larger than that of the curve of *α* = 30°, while the average and minimum values of the latter are smaller. When considering the side incident radar waves, the dynamic RCS curve of *α* = 90° is still significantly higher than the other two curves, because at this time, the sides of the fuselage, the outer wing tips and the cockpit will all provide strong scattering characteristics, while the continuous deflection of the mid-section wing will continue to provide dynamic RCS changes. As the azimuth angle increases from 70° to 80°, the RCS average index increases from – 12 to − 5.08 dBm^2^, where the peak indicator received an increase of 6.7824 dBm^2^, because the curvature of the lower surface of the mid wing is generally small and the surface is smooth, the increase in the azimuth angle will increase the angle between the radar wave and the lower surface of the mid wing, which is not conducive to deflecting the incident wave. Compared with the Z2 mode, the aircraft in the Z3 mode has a slight decrease in the peak and average values of the RCS curve of 90° azimuth, while the peak and average indicators of the RCS curve of the 80° azimuth are slightly increased.

**Table 7:** RCS indicator of the aircraft, *β* = 0°, *f*_RH_ = 6 GHz, *ω*_m1_ = *ω*_m2_ = 0.1963 rad/s, *t* = 0 ~ 4.407 s.

*α* (°)	10	20	30	70	80	90
Mean (dBm^2^)	− 6.5051	− 12.5186	− 18.4253	− 12.0001	− 5.0794	28.0423
Peak (dBm^2^)	− 2.1126	− 7.6584	− 11.1492	− 5.1155	1.6669	43.0769

**Figure 14:**
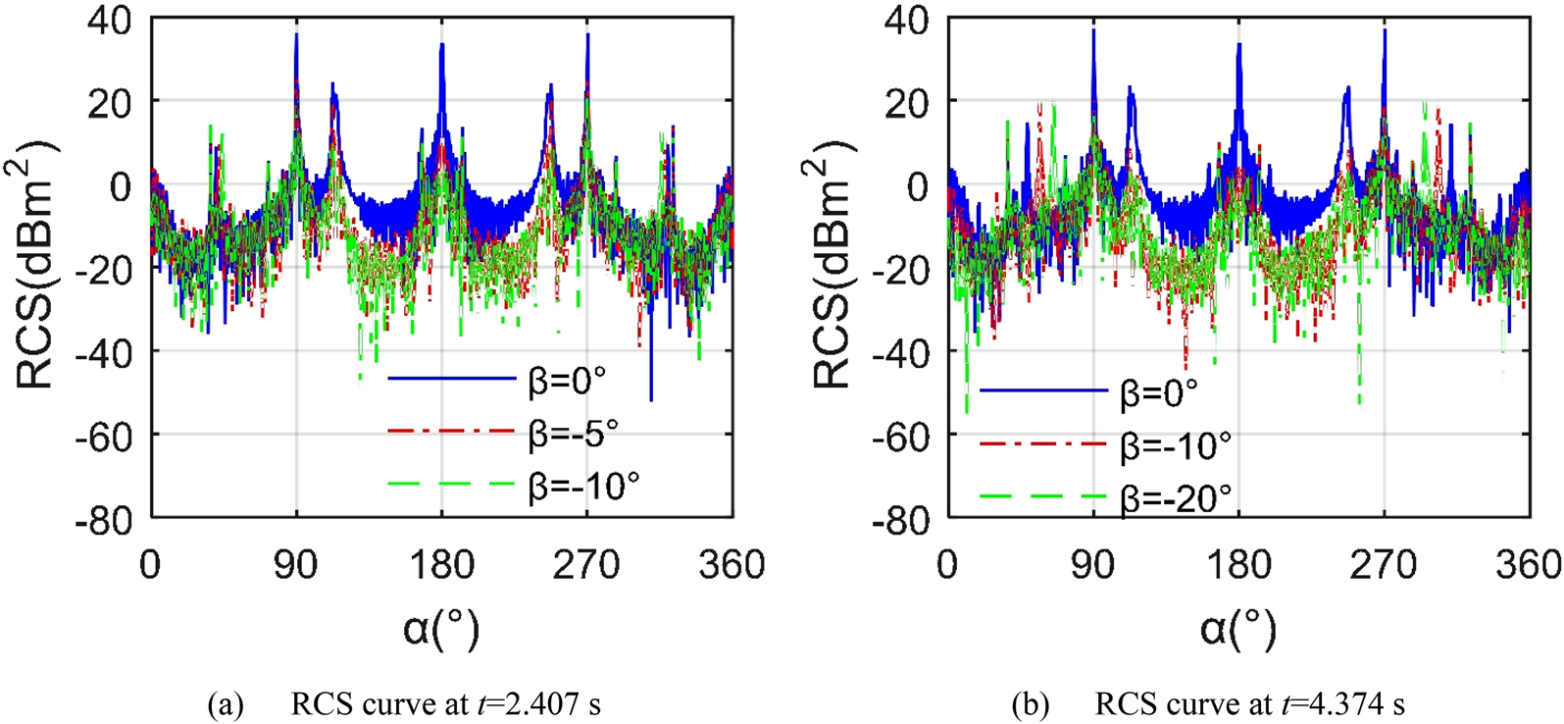
RCS of the aircraft in Z3 mode, *f*_RH_ = 6 GHz, *ω*_m1_ = *ω*_m2_ = 0.1963 rad/s.

Figure 14 shows that in the Z3 mode, the folding deformation of the wings brings significant changes to the aircraft RCS ~ *α* curve, including the mean and the peak value. For the RCS curve at *t* = 2.407 s, the RCS curve with *β* = 0° is significantly higher than the other two, where its average value is as high as 12.68 dBm^2^ as shown in Table 8, because the outer end wing 1 and outer end wing 2 are always kept horizontal, making their outer ends have a poor deflection effect on the incident side waves. When *β* = − 5°, the average RCS index is reduced to 4.59 dBm^2^, while the peak index is reduced by 9.6255 dBm^2^. As the elevation angle continues to decrease to -10°, the peak value continues to decrease, with a magnitude of 20.69 dBm^2^ appearing at 269.8 azimuth. Considering the RCS curve at *t* = 4.374 s, the mid wing deflects upwards by 49.1951°, and the outer wing remains level. As the elevation angle decreases from 0° to − 10°, the mean indicator decreases by 12.441 dBm^2^. When the elevation angle continues to decrease to -20, the change in peak value and average value appears small. These results show that compared with the Z2 mode, the Z3 mode will bring some beneficial improvements to the average and peak indicators of the RCS ~ *α* curve under the current observation conditions.

**Table 8:** RCS indicator of the aircraft in Z3 mode, *α* = 0° ~ 360°, *f*_RH_ = 6 GHz, *ω*_m1_ = *ω*_m2_ = 0.1963 rad/s.

*β* (°)	*t* = 2.407 s			*t* = 4.374 s		
	0	− 5	− 10	0	− 10	− 20
Mean (dBm^2^)	12.6751	4.5932	− 0.0170	13.2345	0.7935	0.8152
Peak (dBm^2^)	36.2149	26.5894	20.6871	37.2017	20.3356	19.8817

### Analysis of Z4 mode

**Figure 15:**
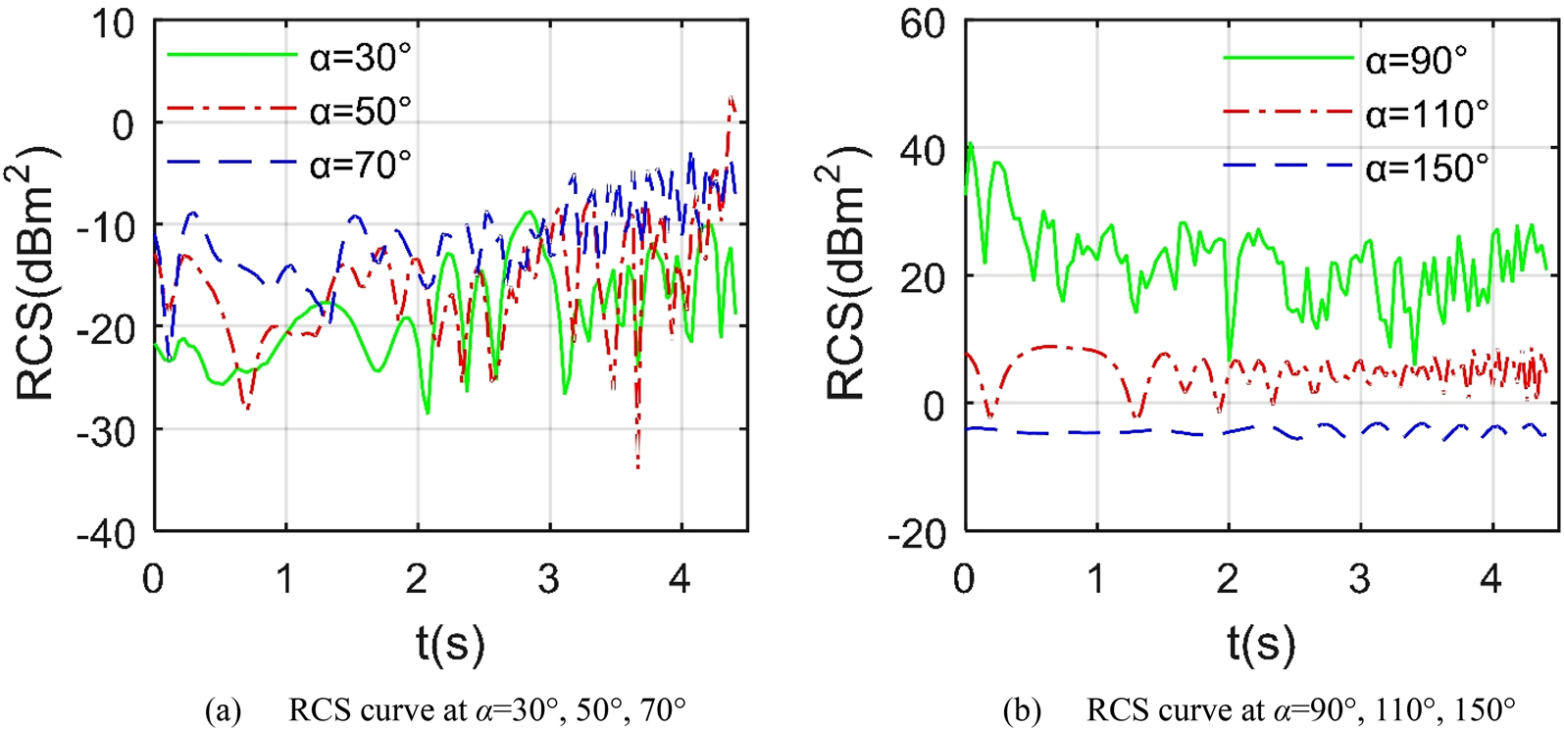
RCS of the aircraft in Z4 mode under various azimuths, *β* = 0°, *f*_RH_ = 6 GHz, *ω*_m1_ = *ω*_m2_ = 0.1963 rad/s.

Figure 15 presents that as the azimuth angle of the incident wave approaches sideways, the average level of the aircraft dynamic RCS curve is increased. For the case of forward direction *α* = 30°, the average RCS is as low as − 16.34 dBm^2^ as shown in Table 9, where the fluctuation range is − 28.62 to − 8.77 dBm^2^. When *α* = 50°, the fluctuation range of the RCS curve has been expanded, and the peak value has also increased significantly. As the azimuth continues to increase to 70°, the average RCS increases to − 9.94 dBm^2^. Consider the positive side incidence, it can be found that the average and peak levels of the RCS curve with *α* = 90° are significantly higher than other curves, because at this time, the side of the fuselage, the cockpit, the wingtip and the upper surface of the outer wing 1 can provide strong scattering sources. When *α* = 110°, the average value of the RCS curve drops to 5.7538 dBm^2^, and the fluctuation range is − 2.936 to 8.841 dBm^2^. Consider the tail direction where *α* = 150°, the fluctuation range is reduced to − 5.844 to − 3.185 dBm^2^, because the dynamic scattering source in the tail direction at this time mainly comes from the trailing edge of the outer wing and the mid wing, these areas all provide weak edge diffraction contribution. In general, compared with the Z3 mode, the average and peak indicators of the dynamic RCS curve of the aircraft under the 90° azimuth in the Z4 mode have been slightly reduced.

**Table 9:** RCS indicator of the aircraft, *β* = 0°, *f*_RH_ = 6 GHz, *ω*_m1_ = *ω*_m2_ = 0.1963 rad/s, *t* = 0 ~ 4.407 s.

*α* (°)	30	50	70	90	110	150
Mean (dBm^2^)	− 16.3428	− 11.5747	− 9.9412	27.2634	5.7538	− 4.4090
Peak (dBm^2^)	− 8.7727	2.5163	− 2.3069	40.8553	8.8411	− 3.1851

**Figure 16:**
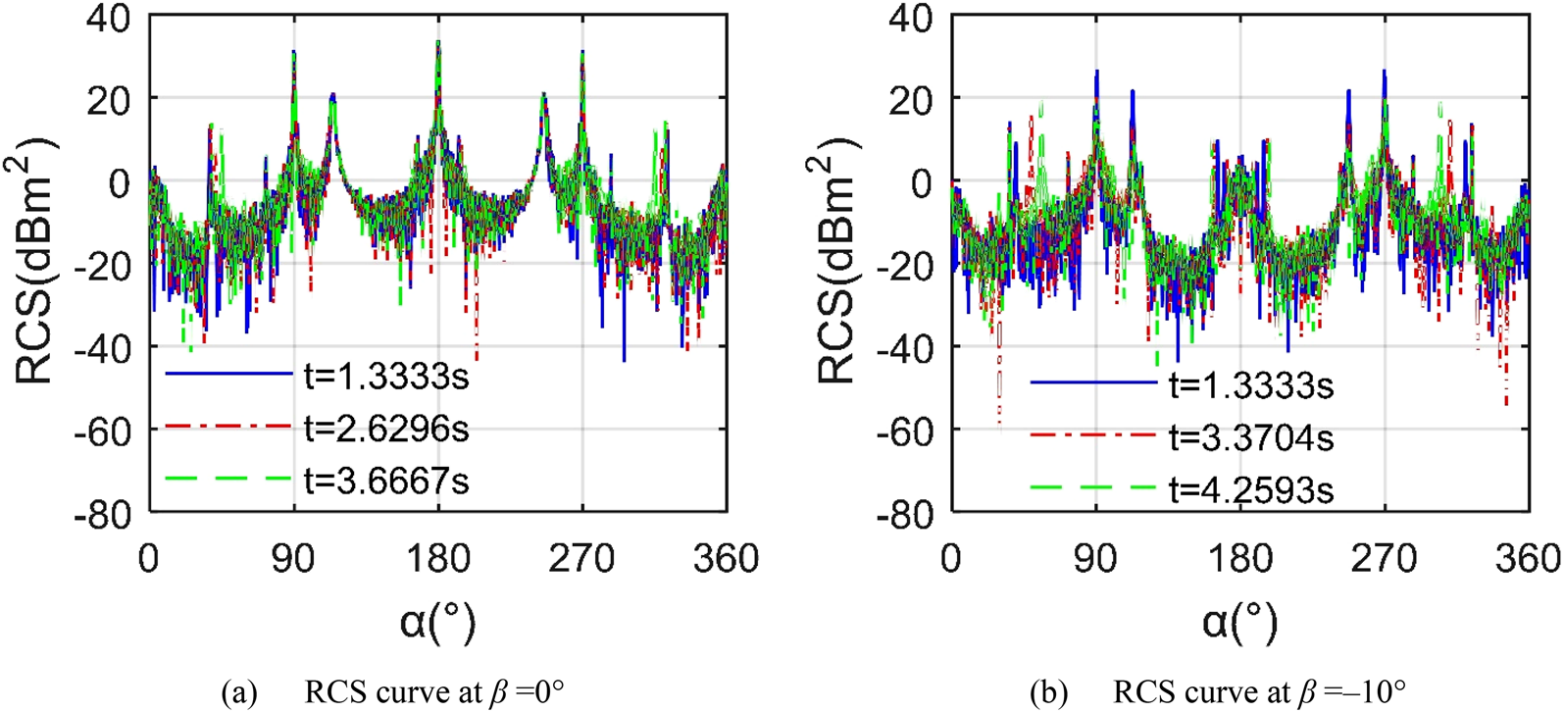
RCS of the aircraft in Z4 mode, *f*_RH_ = 6 GHz, *ω*_m1_ = *ω*_m2_ = 0.1963 rad/s.

Figure 16 indicates that when the elevation angle is reduced from 0° to − 10°, the average and peak indicators of the aircraft RCS in Z4 mode change more obviously. For the RCS curve at *β* = 0°, these three curves are generally similar, while there are obvious differences locally, such as around 40.5 and 319.5 azimuths. When *t* = 2.6296 s, the average value of the RCS curve is equal to 11.42 dBm^2^ as shown in Table 10, and the peak value is 33.76 dBm^2^. As time increases to 3.6667 s, the minimum value of the RCS curve has changed more, while the average and peak indicators have changed less. These changes are mainly attributed to the fact that the sides and the nose of the fuselage provide stable scattering sources for the incident radar waves in the horizontal plane, while the leading and trailing edges of the wing contribute low dynamic scattering, the angle between the wing surface and the horizontal plane is small, which makes the dynamic contribution of the facet weaker. Considering the case of *β* = − 10°, the tail performance of the three RCS curves is significantly reduced. The mean RCS of the curve at *t* = 1.333 s is 5.071 dBm^2^, and the peak is 26.88 dBm^2^ at *α* = 296.8°. When the time is increased to 3.3704 s, the average and peak value have been effectively reduced. As the mid wing and outer wing continue to deflect, the average and peak values of the RCS curve can still maintain a low level, as can be seen in the case of *t* = 4.2593 s. These results indicate that the aircraft deformation in Z4 mode can still maintain a low level of scattering at an elevation angle of − 10°.

**Table 10:** RCS indicator of the aircraft in Z4 mode, *α* = 0° ~ 360°, *f*_RH_ = 6 GHz, *ω*_m1_ = *ω*_m2_ = 0.1963 rad/s.

*t* (s)	*β* = 0°			*β* = − 10°		
	1.3333	2.6296	3.6667	1.3333	3.3704	4.2593
Mean (dBm^2^)	11.6318	11.4199	11.6341	5.0710	1.5495	1.3879
Peak (dBm^2^)	33.7593	33.7582	33.7526	26.8809	21.0381	20.5785

## Conclusion

Based on the presented HGMT method, the electromagnetic scattering characteristics of the morphing aircraft in the four Z-folding modes are studied and discussed. Through these investigations and analyses, this manuscript can draw the following three conclusions (Supplementary information S1):

1. For a given incident wave, the deflection of the outer wing alone will bring significant dynamic changes to its RCS, while in Z1 and Z2 folding modes, the maximum peak of the dynamic RCS of the aircraft under the lateral azimuth is significantly higher than that under the head azimuth.
2. Under the condition of positive lateral incident wave, the given folding deformation of the wing in Z3 mode can significantly reduce the dynamic RCS level of the aircraft, where the fluctuation range of the RCS curve after the maximum peak also appears relatively stable, while the maximum peak is reduced compared with Z2 mode.
3. In Z4 mode, the maximum peak of the dynamic RCS curve of the aircraft under the positive lateral azimuth is further reduced, where at the given negative elevation angle, the aircraft can still gain good stealth characteristics, while the dynamic range of the RCS after the maximum peak becomes larger compared with other modes.

## Supplementary Information

Supplementary Information.
