# Supplementary material for: Z-folding aircraft electromagnetic scattering analysis based on hybrid grid matrix transformation
Source: Sci Rep. 2022 Mar 15;12:4452. doi: 10.1038/s41598-022-08385-9 (PMC8924198; doi:10.1038/s41598-022-08385-9)
Supplement: Supplementary file 1 — Supplementary Information. [file 41598_2022_8385_MOESM1_ESM.docx]

**Appendix A**

In order to study the influence of the elastic deformation of the wing 1 on its RCS, the maximum deformation among all the element nodes is introduced here:

$E_{\mathrm{dm}}=\max\left\{E_{\mathrm{d}}\left(i\right)\left|i=1,2,...,N_{\mathrm{FN}}\right.\right\}$ (A1)

where *N*_FN_ represents the number of all nodes of the wing facets, *E*_d_ is the amount of elastic deformation at the node, *E*_dm_ is the maximum elastic deformation. The wing deforms along the spanwise direction, and the deformation set at the wing tip reaches the maximum. The elastic deformation at each node is calculated according to the curve characteristics of the right half of the quadratic function with the opening upward:

$E_{\mathrm{d}}\left(i\right)=A_{\mathrm{e}1}\left(y\left(i\right)-y_{\mathrm{n}1}\right)^{2}+B_{\mathrm{e}1}$ (A2)

where *A*_e1_ and *B*_e1_ are the coefficients of this quadratic function, *y*_n1_ is the *y* coordinate value of the node closest to the coordinate origin. Noting that:

$y_{\mathrm{d}1}=y_{\mathrm{m}1}-y_{\mathrm{n}1}$ (A3)

$A_{\mathrm{e}1}=\frac{E_{\mathrm{dm}}}{y_{\mathrm{d}1}^{2}}$ (A4)

where *y*_m1_ is the *y* coordinate value of the node farthest from the coordinate origin. Therefore, the vertical coordinates of each bin node need to be updated:

$Z\left(i\right)=z\left(i\right)+E_{\mathrm{d}}\left(i\right)$ (A5)

where *Z*(*i*) represents the new vertical coordinate of the facet node.

The effect of elastic deformation on the RCS of wing 1 is shown in Figure A1, where the blue dotted line represents the RCS without considering elastic deformation, and the red solid line represents the RCS considering elastic deformation. At this time, wing 1 deflects downward by 0.6667°, where the mean value of the blue line is 8.9599 dBm^2^ and the peak value is 39.8681 dBm^2^. Although the maximum deformation of the wing tip reaches 0.56 m, the mean RCS of the red line is still 8.9554 dBm^2^ and the peak is 39.8676 dBm^2^. It can be considered that the two curves are generally similar, including curve shape, peak and mean level. These results show that the effect of the current elastic deformation on the RCS of the wing can be ignored.


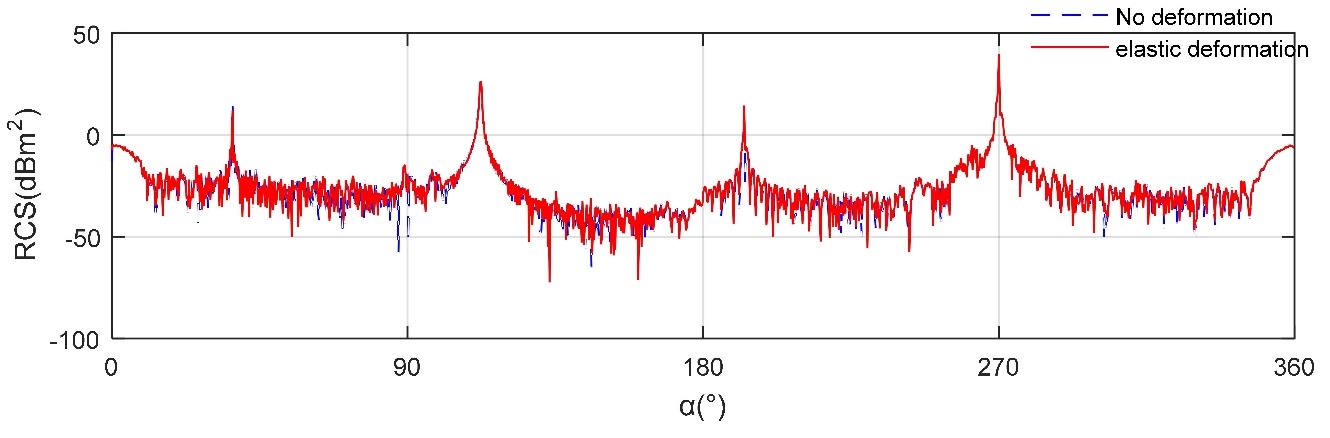


Fig. A1 Effect of elastic deformation on RCS of wing 1, *E*_dm_=0.56 m, *f*_RH_=10 GHz, *β*=0°, *ω*_r1_=0.3142 rad/s, *t*=0.037 s.

The effect of elastic deformation of wing 1 is shown in Figure A2, where the maximum elastic deformation is set to 0.68 m. At the current moment, the wing has not deflected. The influence of elastic deformation on the characteristics of wing surface is obvious. When the elastic deformation is not considered, it can be found that the whole wing remains flat and the performance is generally blue. When elastic deformation is considered, the tip of the wing tilts upward because the maximum deformation here is set to a positive value. From the bottom to the top of the wing, the color changes from blue to yellow, where the color here is used to indicate the ordinate size of the node.


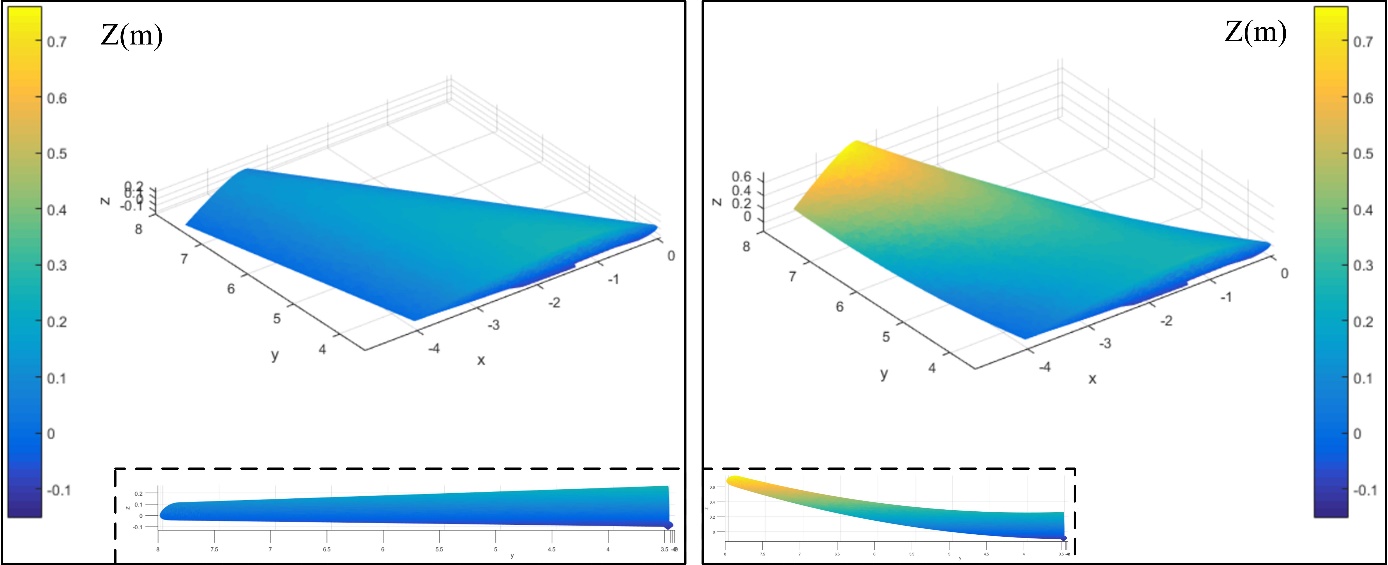


| (a) No elastic deformation | (b) Elastic deformation |
| --- | --- |

Fig. A2 Effect of elastic deformation on the wing 1, *E*_dm_=0.68 m, *ω*_r1_=0.3142 rad/s, *t*=0 s.
